# Frailty assessment utilization around the globe–a systematic review

**DOI:** 10.1016/j.tjfa.2025.100088

**Published:** 2025-10-02

**Authors:** Samantha Gaston, Elle Billman, Lichy Han, David Drover

**Affiliations:** a Stanford University School of Medicine, 300 Pasteur Drive, Dept Of Anesthesia H3580, Stanford, California 94305-2200; b Icahn School of Medicine at Mount Sinai; c Stanford University School of Medicine; d Professor of Anesthesiology, Perioperative, and Pain Medicine, Stanford University School of Medicine

**Keywords:** Frailty index, Frailty assessment, Frailty instruments, Systematic review, Geriatric participants

## Abstract

**Background:**

Recent expert guidelines recommend that frailty assessments (FAs) encompass physical, functional, cognitive, social, and mental health domains. This systematic review examines FAs administered globally between 2015 to 2022 in geriatric participants (65 years and older) to characterize the parameters used to assess frailty.

**Methods:**

Following PRISMA guidelines, we screened 3,859 articles and included 202 in the final analysis. FA parameters were coded into 45 health-related categories defined by the authors to evaluate the domains most frequently used.

**Results:**

Across 39 countries, 291 FAs were identified, with an average number of 17.36 parameters per instrument. Of the 4,995 total parameters analyzed, 22.32 % assessed functional health or physical performance. Cognitive, mental, and social health were assessed by only 6.09 %, 6.35 %, and 5.01 % of parameters, respectively.

**Conclusions:**

FAs overwhelmingly measure functional and physical health parameters with limited attention to cognitive, mental, and social domains. This imbalance suggests that instruments may fall short of capturing the multidimensional nature of frailty as recommended by recent guidelines. By cataloging current FAs, their components, and the degree to which they reflect comprehensive frailty definitions, this review highlights the need for further research and refinement of FAs to ensure accurate, holistic assessment across diverse clinical settings.

## Introduction

Although there is currently no universal consensus on how to define frailty [[1], [2], [3], [4], [5]], frailty assessments (FAs) are widely recognized as clinically valuable tools. They have been associated with increased risk of disability, falls, hospitalization, delirium, institutionalization, morbidity, and mortality in postoperative patients [[5], [6], [7], [8], [9], [10]], as well as an increased need for assistance in daily activities [6,[11], [12], [13], [14], [15]]. Regular frailty screening is recommended in individuals over the age of 75 [16,17] and, within perioperative medicine, guidelines advocate for the use of FAs to identify patients at risk of poor surgical outcomes [11,12,[18], [17], [19], [20], [21], [22],^23^]. Despite these recommendations, frailty assessments remain underutilized. Contributing factors include limited clinical time for administration [24] and the absence of standardized guidance on which instruments most accurately capture frailty. This lack of consensus stems from differing conceptualizations and operationalizations of FAs [1,5,20,24] which has driven the development and testing of many different FAs [5].

Most assessments are grounded in one of two approaches: phenotypic or deficit accumulation models [5,9,10]. The phenotypic approach led to the development of the Fried Phenotype which defines frailty as a syndrome identified by five physical criteria–unintentional weight loss, weakness, self-reported exhaustion, slow walking speed, and low physical activity–characterizing individuals as not frail, pre-frail, intermediate, or frail [25]. In contrast, the deficit accumulation model conceptualizes frailty as a dynamic state that is a sum of deficits across multidimensional domains of health which are converted to ratios that reflect the severity of frailty [26,27].

Between 1997 and 2009, the phenotypic approach was widely favored because of its more-easily-captured parameters [28]. However, this model has been criticized for neglecting to capture comprehensive elements of frailty related to psychological, cognitive, and social health. Comparative studies have found that phenotypic and deficit accumulation models yield differing frailty prevalence rates within the same populations, with the phenotypic approach generally identifying fewer individuals as frail [[28], [29], [30], [31], [32]]. This discrepancy has led to concerns that phenotypic models may underestimate the prevalence of frailty. Nevertheless, a recent systematic review evaluating studies published between 2009 to 2018 found that phenotypic-based assessments remain the most utilized [17].

As it stands, there does not yet exist a definitive definition of frailty. However, in 2013, an expert panel convened through the Frailty Operative Definition-Consensus Conference Project to attempt to define frailty in a clinical setting [1]. Rodriguez-Mañas et al proposed that frailty be recognized as a multidimensional syndrome and recommended its assessment include physical performance, (e.g., gait speed and mobility), nutritional status, mental health, and cognition [1]. Although no consensus was reached on specific diagnostic procedures when it comes to frailty, this multi domain approach offers a potential framework that may be clinically useful. The panel also concluded that no single biomarker sufficiently captures frailty and called for further research to identify the specific combination of clinical and laboratory biomarkers that can be used for the diagnosis of frailty [1].

Recently, the World Health Organization utilized its Integrated Care for Older People (ICOPE) framework to conceptualize frailty not only as physical decline, but also as a progressive loss of “intrinsic capacity” [33]. This “intrinsic capacity” encompasses cognitive health, psychological well-being, mobility, sensory function (i.e. vision and hearing), and the ability to maintain social relationships and contribute to society. The ICOPE ideology provides a foundation for understanding frailty as a multidimensional concept and underlines the need for assessments that go beyond physical symptoms to capture cognitive, psychological, functional, nutritional, and social domains. This multidimensional perspective aligns with recent expert recommendations and reflects the evolving understanding of frailty as a complex and dynamic condition that requires holistic assessment and care strategies.

Despite these expert recommendations, it remains unclear whether multidimensional FAs are the optimal metric of frailty across varied clinical contexts. Since the 2013 conference, research has increasingly focused on FA development [19], the assessment of construct validity of FAs, the prevalence of frailty in various patient populations [19,[34], [35], [36], [37], [38], [39], [40]], the effects of interventions on frailty status [19,[41], [42], [43], [44]], and outcomes associated with frailty [[45], [46], [47], [48]]. However, further research may be needed to determine whether newer FAs adequately capture the comprehensive domains outlined by experts.

The purpose of this systematic review is to examine frailty assessments used in studies published across the globe between 2015 to 2022 in geriatric subjects, and to evaluate the domains captured by assessments. In doing so, we aim to characterize the variables of health used to capture frailty, assess their alignment with expert recommendations, and provide a resource for researchers to refer to when considering what parameters are frequently captured in FAs.

## Methods

**Fig. 1:**
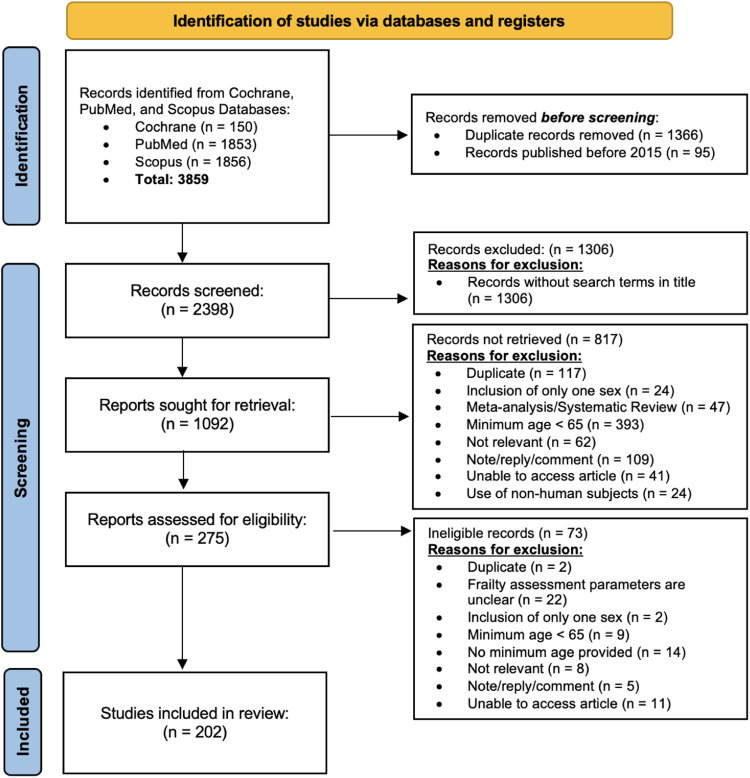
Preferred Reporting Items for Systematic Reviews and Meta-Analyses (PRISMA) guidelines.

This systematic review was conducted in accordance with the Preferred Reporting Items for Systematic Reviews and Meta-Analyses (PRISMA) guidelines (Fig. 1) [49]. A comprehensive electronic search was performed across three databases–Cochrane, Scopus, and PubMed–to identify studies published between January 2015 and September 2022. Studies were included if they contained the following search terms in their title: “frailty index”, “frailty indexes”, “frailty indices”, “frailty assessment”, “assessment of frailty,” “frailty analysis,” “frailty indicator,” “frailty questionnaire”, and “frailty instrument.” Duplicate papers were excluded. Although the search terms were applied consistently across databases, 1,306 papers were excluded post hoc for not containing one of the predefined search terms in their titles. Two authors (EB and SG) screened the abstracts for relevance and eligibility. Prior to formal screening, they calibrated their approach to ensure consistency. The authors independently reviewed the studies, flagged any papers with questionable eligibility, and discussed any discrepancies. When uncertainty remained, the authors jointly evaluated the full text to determine inclusion.

To focus the review on relevant clinical populations, we excluded studies involving participants younger than 65 years of age, reflecting the definition of a geriatric population in many countries [2]. It was our goal to assess papers that applied the stated frailty assessment(s) to a participant population, so meta-analyses, editorials, comments/replies, et cetera, were excluded. To ensure inclusivity and generalizability, studies were excluded if they were restricted to only one sex or gender. To gather information on the utilization of FAs on a variety of participant populations, papers were included regardless of participants’ race/ethnicity, health status/condition, or geographic location. Additional exclusion criteria included non-human research subjects, a lack of public or institutional access to the full text, or irrelevance to the aims of this study. Papers were deemed irrelevant if they focused on claims-based algorithm development and/or modeling techniques, device development, or did not test FAs with participant data. Finally, papers published in all languages were accepted so long as an English translation was available or Google Translate could provide an adequate translation. Of the 275 full-text articles reviewed, 73 were excluded for not meeting inclusion criteria. Ultimately, 202 papers were included in the final data extraction process.

During data extraction, the following variables were captured: study title, author(s), year of publication, country in which data collection occurred, sample size, minimum participant age, mean or median participant age, participant health condition (if provided), FA(s) used within the study, parameters included in the FAs, and average/estimated time needed to complete the given FAs. During the data extraction process, papers were deemed ineligible if the FA parameters were not explicitly stated and/or if they were not clearly delineated, if a minimum age was not provided by a study, or if the papers met any of the previous exclusion criteria (e.g. including subjects under 65 years of age).

To categorize the FA parameters, the authors identified 45 categories that spanned physical, mental, social, cognitive, and functional domains. The categories were initially derived from organ system groupings [50] and subsequently expanded to capture frequently measured elements–such as nutrition and polypharmacy–to provide a specific and operationalized summary of parameters. Each FA parameter was assigned to 1 of 45 categories based on the primary domain of health it assessed. Table 1 presents the complete list of the parameter categories, subcategories, and examples of parameters assigned to a given category. Parameters that encompassed multiple domains (e.g. “renal, urological, or genital disorders”), could not be clearly categorized (e.g. “do not resuscitate), or were too infrequent to warrant a separate category were classified under Category 17: “Other.” The Clinical Frailty Scale (CFS), a nine-point scale incorporating elements of disease, activities of daily living, and independence, was also assigned to Category 17 due to its composite nature, which precluded disaggregation into individual parameter categories (Table 2).

**Table 1:** Parameter categories and percentages.

**Table 2:** Frequency and type of frailty instruments administered per country. Key: EFS - Edmonton Frail Scale, GFI - Groningen Frailty Indicator (GFI), TFI - Tilburg Frailty Index, GFST - Gerontopole Frailty Screening Tool, Fried – Fried and modifications of Fried, FTS – Frailty Trait Scale, U – deficit accumulation-based FI, O – other (frailty instruments were assigned as ‘other’ if they were administered in three or fewer different countries.

Country	# of papers	# of FAs	mFI 11*	mFI 5*	FI-CGA*	Fried*	FI-ED*	CFS*	FI-LAB*	TFI*	FRAIL*	eFI*	SHARE FI*	GFI*	EFS*	GFST*	CHS*	FTS*	U*	O*
**Australia**	14	18	33.3	5.5	11.1	11.1													27.8	11.1
**Austria**	1	1					100													
**Belgium**	1	1					100													
**Brazil**	1	1																	100	
**Canada**	12	16	12.5	6.25	12.5	12.5		18.8											6.3	12.5
**China**	19	25	8		4	16			8		4	4		4			4		32	16
**Croatia**	1	1								100										
**Denmark**	3	4								50										50
**England**	3	5		20				40				40								
**Finland**	1	1																		100
**France**	6	22			4.5	18.2		9.1			9.1		9.1			9.1		18.2	13.6	9.1
**Germany**	10	22			22.7	18.2	4.5	9.1	9.1		4.5	4.5		4.5					13.6	9.1
**Greece**	3	4				25		25		25										25
**Iceland**	1	1					100													
**India**	2	2				50	50													
**Ireland**	1	1											100							
**Italy**	16	25				16		12		4			8			8		16	20	16
**Japan**	10	14	7.2												7.1			7.1	21.4	57.2
**Korea (unspecified)**	8	11	9.1		9.1	9.1					18.2							9.1	9	36.4
**Lebanon**	1	1												100						
**Lithuania**	1	1													100					
**Mexico**	1	1				100														
**Netherlands**	13	15				6.7				46.7		6.7		26.7					6.5	6.7
**New Zealand**	2	2													50				50	
**Norway**	1	1																		100
**Poland**	6	24				12.5		12.5		12.5	16.7		8.3		4.2	8.3		16.7		8.3
**Portugal**	1	1								100										
**Saudi Arabia**	2	7								16.7	16.7									66.6
**Scotland**	1	2		50				50												
**South Korea**	4	6				16.7			33.3										33.3	16.7
**Spain**	16	36	2.8			13.9		5.6		8.3	11	2.8	8.3		2.8	5.6		13.9	8.3	16.7
**Sweden**	1	1					100													
**Switzerland**	1	1																	100	
**Taiwan**	2	4									25						25			50
**Thailand**	1	2				50			50											
**Turkey**	6	7		14.3		14.3				28.5					14.3					28.6
**United Kingdom**	9	23				8.7		13		4.3	8.7	13	8.8			8.7		17.4	8.7	8.7
**USA**	45	58	6.9	6.9	1.7	17.2		5.2	1.7		5.2	3.4			1.7				15.5	34.6

⁎ All values presented in these columns are displayed as percentages. The denominator for each calculation is the total number of papers published in a given country. The numerator for each calculation is the frequency of individual frailty instruments administered in a country.

Functional health/physical performance (Category 14) included parameters that assessed activities of daily living as well as manifestations of physical health including parameters assessing exercise; level of autonomy in tasks such as cooking, managing finances, transportation, personal hygiene, et cetera; chair stands; unspecified strength; unspecified slowness; and balance. This parameter also included reported exhaustion and fatigue pertaining to physical and psychological functioning. With Category 14, Functional Health/Physical Performance, we categorized parameters that were physical manifestations of one’s physical health within the functional health category. For example, while conditions like arthritis were categorized under specific organ systems (e.g. musculoskeletal–Category 1), resulting functional limitations (i.e. difficulty climbing stairs) were categorized separately under functional health.

Given the nature of this systematic review and the focus on the content and distribution of frailty assessment parameters rather than their outcomes, a formal risk of bias assessment was not performed. However, efforts were made to minimize bias by implementing clearly defined inclusion and exclusion criteria, using dual independent abstract screening and full-text review, and excluding studies lacking participant-level data or clearly delineated FA parameters. Studies were excluded if they did not provide a minimum participant age, used claims-based assessments without clinical data, or did not administer FAs to human subjects. The reproducibility of parameter categorization was strengthened by calibrating screening procedures prior to data extraction and by clearly operationalizing 45 parameter categories across the five health domains.

Following data extraction, the included studies were analyzed to determine the average participant age, average sample size, number of parameters per FA, distribution of parameter categories within each FA (i.e., which categories were included in each index), and frequency of each category across the entire dataset.

## Results

An initial search of the Cochrane, Scopus, and PubMed databases yielded 3859 papers. After applying inclusion and exclusion criteria, 202 papers were included in the final analysis. The average age of participants across studies was 77.22 ± 4.42 years. The average sample size was 105,100 participants, with a median sample size of 482, indicating highly skewed data; 37 papers were identified as outliers in the sample size analysis. Across the studies, 291 distinct FAs were identified, with 52 papers utilizing more than one FA, encompassing a total of 4995 parameters. The average number of parameters used per FA was 17.36 ± 17.37, while the average number of parameters used *per study* was 24.9 ± 20.46.

**Fig. 2:**
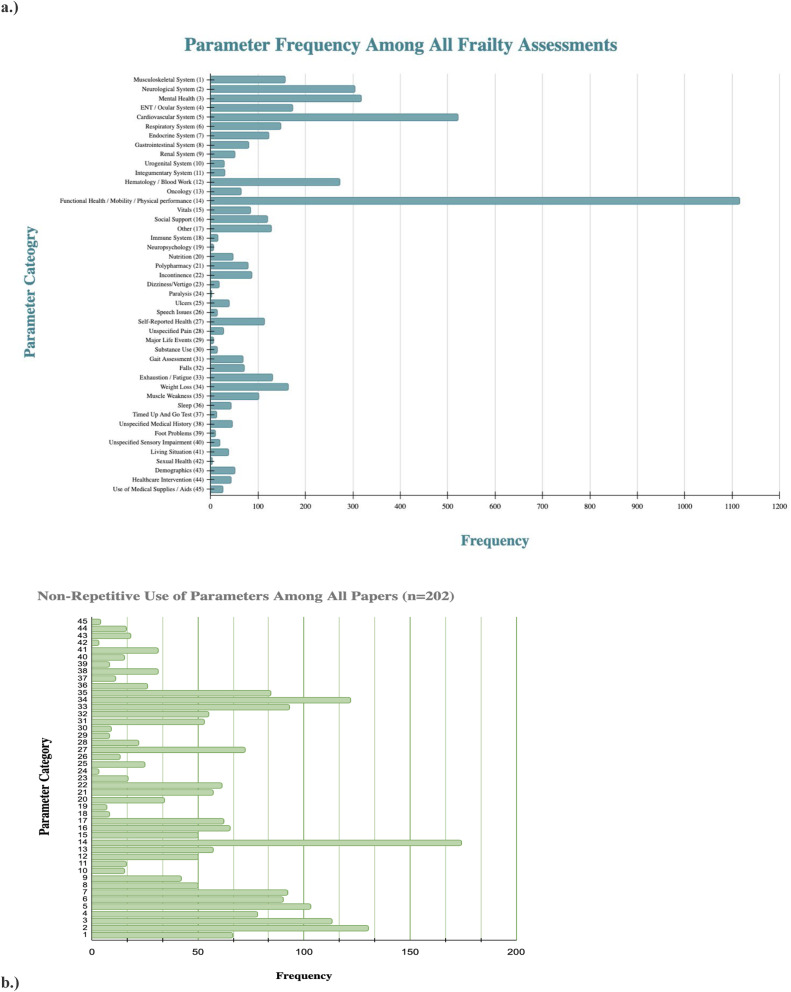
**a**). These figures display the distribution of all parameters utilized across 202 papers (for a total of 4995 parameters) across the derived 45 categories **b.)** This figure displays the non-repetitive frequency of the 45 categories among the 202 papers. For example, if one paper had 10 parameters, five of which went under the Neurology category and the other five were classified as Mental Health, this paper would be said to include 2 categories.

Using our defined parameter categories, Functional Health/Physical Performance (Category 14) was the most frequently represented category, comprising 22.32 % of the total 4995 parameters. This was followed by Cardiovascular Health (Category 5) at 10.43 %, and Psychological Health/Mental Health (Category 3) at 6.35 % (see Fig. 2a). When assessed at the study level (i.e., whether a study included at least one parameter from a given category, Category 14 appeared in 84.14 % of studies, followed by Neurological Health (Category 2) at 64.26 % and Weight Loss (Category 34) at 60.40 % (see Fig. 2b).

**Fig. 3a:**
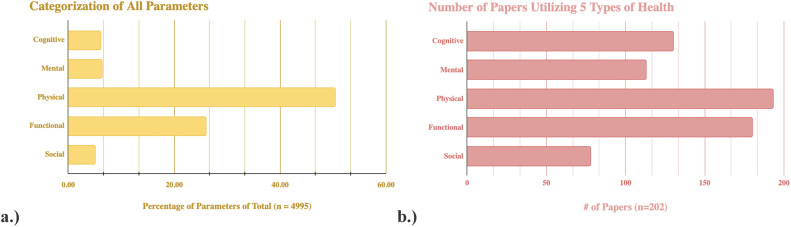
The authors further grouped the 45 categories into 5 distinct types of health: cognitive, mental, physical, functional, and social health. Some categories, such as Category 17 (other) could not be accurately grouped into one of these 5 types of health and were not included in the analysis. The figure above demonstrates the distribution of parameters among these different types of health. **b.** This figure demonstrates the distribution of papers that included parameters within the 5 types of health listed above.

Utilizing the definitions of frailty delineated by Rodríguez-Mañas et al [1]–cognition (corresponding to Category 2), mental health (Category 3), mobility (Category 14), nutritional status (Category 20), and gait speed (Category 31)–only 5.45 % of 202 papers included all five domains among their total parameters. An additional 16.83 % included four domains, 33.17 % included three, 18.81 % included two, and 18.81 % included only one. Notably, 6.93 % of the 202 papers did not contain any of these domains (see Fig. 3). This indicates that FAs including multidimensional domains remains limited despite growing awareness of the importance of holistic frailty assessments.

Further categorization of the parameters into five overarching health domains showed that 193 studies included physical parameters (Categories 1, 4-13, 18, 20-28, 32-34, 36, 39, 40), 113 included mental health (Category 3), 78 included social health (Categories 16, 41, 43, 44), 130 included cognitive health (Category 2), and 180 included functional health (Categories 14, 31, 35, 37). Of the total 4,995 parameters, 50.41 % pertained to physical health, 6.35 % to mental health, 5.01 % to social health, 6.09 % to cognitive health, and 25.95 % related to functional health (see Fig. 3b).

A notable percentage of parameters (classified as Category 17, “Other”) include composite tools such as the Clinical Frail Scale (CFS), which could not be clearly categorized into a single health domain. CFS was used in 19 studies, and this category accounted for 2.56 % of all parameters. The inclusion of composite or non-specific assessments highlights the interpretive challenges posed by multidimensional instruments and the need for greater domain clarity and specificity in FA design.

The six most frequently used FAs were Fried’s Frailty Phenotype (38/202 studies) [25], Clinical Frail Scale (19/202) [51], Tilburg Frailty Indicator (19/202) [30], 11-item modified Frailty Index (mFI) (14/202) [52], electronic Frailty Index (eFI) (11/202), and Comprehensive Geriatric Assessment (11/202). The use of eFIs, a tool that pulls patient data from electronic health records (EHR), illustrates the increasing practice of utilizing existing clinical data for large-scale, objective frailty screening. eFIs were used across studies in Europe, North America, and Asia, demonstrating their increasing global prominence in health systems aiming for efficient frailty monitoring on a population level.

However, it is worth noting that 35 FAs were unspecified (referred to generically), 43 FAs were only utilized once, and 67 FAs were used, at most, twice (i.e. frequency ≤ 2). This high variability in FA usage reflects the lack of an international consensus on standardized frailty assessments and demonstrates the diversity in healthcare practices and values.

Studies spanned 39 countries, with the largest representation from the United States of America (19.82 %), China (8.37 %), Spain (7.05 %), and Italy (7.05 %). FA utilization patterns varied by region. The Tilburg Frailty Indicator appeared in studies from Asia, South America, Europe, and the Middle East, but not North America or Australia. FI-Lab instruments and eFIs, both increasingly common frailty instruments, were administered in Asia, Europe, and North America. The SHARE Frailty Instrument was exclusively used in Europe and was developed as an instrument to assess frailty in primary care settings [53]. Similarly, the Gérontopôle Frailty Screening Tool (GFST) was developed for general practitioners in France to identify frailty but is now being used outside of France in other European countries [54]. Lastly, the Kihon Checklist, which was developed in Japan, was administered in Japan and Spain. The Kihon Checklist is culturally specific to Japan and was administered in a study in Spain to determine its validity in a new population [55]. These findings demonstrate that while some tools (i.e. Fried’s Phenotype, mFI, eFI) have been implemented internationally, others remain regionally concentrated and culturally specific. This diversity likely reflects both structural healthcare differences and sociocultural attitudes toward aging, independence, and social support.

We also assessed how frequently FAs included parameters related to cognitive, mental, and social health across the top five contributing countries: the United States, Spain, Italy, China, and Australia. Cognitive and mental health were similarly represented in studies from the U.S., Italy, and Australia. Spain had the highest frequency of FAs incorporating social health parameters and led in inclusion of cognitive health parameters. Italy had the highest frequency of FAs including mental health assessments. In contrast, none of the studies from China included parameters related to social health, although cognitive and mental health were assessed at similar frequencies. This cross-country variation may reflect cultural priorities in health assessments, differing healthcare practices in delivering such assessments, or disparities in access to multidomain assessment tools.

## Discussion

Frailty is strongly associated with advancing age, poor health outcomes, and adverse events such as hospitalization, disability, and mortality. Consequently, frailty assessments (FAs) have become valuable tools for identifying at-risk individuals and guiding patient management, especially in the context of a rapidly aging global population. This systematic review aimed to inventory the variety and content of FAs used worldwide between 2015 and 2022.

Historically, frailty assessments have emphasized physical and functional domains, as exemplified by Fried’s Frailty Phenotype. Our review confirms that this trend persists: among 202 studies, 193 (95.5 %) included physical health parameters and 180 (89.1 %) included functional health parameters. Over half (50.41 %) of all 4,995 parameters pertained to physical health, and 25.95 % to functional health.

While these domains are integral to frailty, growing evidence underscores the importance of including mental, cognitive, and social dimensions in frailty assessments. Recent research has demonstrated that depression is highly prevalent in frail individuals (reported in up to 46.5 %) and may increase the risk of frailty [56,57]. Cognitive frailty is associated with elevated risks of falls, disability, and hospitalization [58]. Similarly, social vulnerability–including loneliness, social isolation, and inadequate support–has been linked to more rapid frailty progression [59], [60], [61]. Despite these associations, we found only 6.09 %, 6.35 %, and 5.01 % of all 4995 parameters addressed cognitive, mental, and social health, respectively. This may indicate that parameters imperative to diagnosing frailty are not being adequately addressed by FAs. While we acknowledge that physical and functional health are perhaps more easily observed and/or quantified by patients and healthcare professionals, cognitive, mental, and the social determinants of health cannot be excluded in the identification of frailty.

The World Health Organization’s Integrated Care for Older People (ICOPE) uses the idea of “intrinsic capacity” which is the combination of physical, mental, cognitive, sensory, and social health [33]. ICOPE emphasizes the need for “stepped care” in which short screenings can identify individuals at risk [33]. These screenings may then be followed by detailed multidomain assessments to inform personalized interventions. This approach highlights the importance of FAs are guides to comprehensive care.

It is essential to distinguish, however, between frailty screening tools and comprehensive geriatric assessments (CGAs). Screening tools are typically brief and domain-limited to ensure ease of administration and diagnostic accuracy in various settings. As noted in ICFSR Clinical Practice Guidelines [62], positive screening results should prompt a multi-domain clinical assessment to inform diagnosis, risk stratification, and care planning. This stepped approach mirrors ICOPE’s model and supports this two-step practice of first completing a risk identification and then a more in-depth multidimensional evaluation. Thus, the lack of comprehensiveness in many tools may reflect their role in initial screening rather than their failure as holistic instruments.

Nevertheless, our findings raise concern about the overreliance on narrow-domain instruments without clear pathways for follow-up assessments. Only 5.45 % of studies included instruments that spanned all five core domains outlined by Rodríguez-Mañas et al [1]–cognition, mental health, mobility, nutritional status, and gait speed. While 16.83 % of studies included four of these domains, nearly 44 % included one or none, which may limit the effectiveness of frailty identification and subsequent management in clinical practice. This is clinically significant in that limited assessments may fail to identify at-risk patients or misinform risk evaluation which may delay intervention. While research efforts continue to investigate what variables are the most valid predictors of frailty and while the optimal way to measure frailty likely varies depending on setting, researchers developing FAs should be certain to use parameters that capture multiple domains of health.

The variability and often ambiguous nature of certain parameters further complicate matters. For instance, Bessa et al gives the example of parameters asking patients about loneliness–assessing loneliness could be used to discover mental health concerns or rather the amount of social support one has in their life [63]. Likewise, the Kihon checklist asks, “Do you sometimes visit your friends?” This could be measuring if a person has a social group to visit but could also assess if a person is physically able to leave their home and navigate transportation to visit their friends [55]. Finally, the Tilburg assessment asks, “Have you felt down during the last month [30]?” An affirmative response to this question could be due to a multitude of reasons including mental health, social health, or physical health challenges. Improved specificity in parameter design is needed to ensure accurate domain assessment. Future FAs should strive not only for domain inclusivity but also for conceptual clarity, ensuring that questions are aligned with their intended health construct.

Additionally, cultural context must also be considered. Complex domains such as social health, independence, and self-rated health may be understood differently across cultures. For example, the concept of independence may look different depending on cultural norms. It may be necessary to have localized adaptations of FAs to maintain validity and cultural relevance.

Our findings also highlight the increasing usage of electronic frailty indices (eFIs), with data taken from electronic health record (EHR) data such as ICD-10 codes, lab values, and medication records to estimate frailty objectively. eFIs are advantageous in that the data is readily accessible, they can be monitored in real-time, and they may have more standardized units across institutions (such as lab values). However, they may not capture more complex domains such as cognitive and/or mental health, emphasizing the need for comprehensive assessments in these areas.

Given our findings, we propose several policy recommendations. First, healthcare systems should adopt a standardized, multidomain frailty screening assessment that follows expert recommendations such as those outlined by ICOPE. Second, national healthcare infrastructures should integrate FAs into EHR systems to allow for long-term frailty monitoring and appropriate intervention depending on a given frailty assessment. Third, clinical guidelines should follow a two-step process of a short risk assessment followed by a more comprehensive frailty evaluation. These recommendations are already being implemented in several countries. For example, the United Kingdom’s National Health Service has required the use of an eFI in their primary care EHR [64]. Japan uses the Kihon Checklist in communities to provide long-term care prevention screening [65]. Finally, Korea’s 2021 Frailty Clinical Practice Guidelines, developed by the Korean Academy of Family Medicine, include standardized recommendations for frailty diagnosis in primary care settings [66].

The utility of FAs ultimately lies not only in their diagnostic capacity but also in their ability to predict adverse outcomes, inform individualized care plans, and guide resource allocation. Tools that fail to assess critical domains such as cognition or social or social support may miss important predictors of health decline and healthcare utilization. The goal may not be comprehensive assessment at the point of screening, but to ensure that screening triggers appropriate, in-depth evaluations across all relevant domains.

Lastly, as the global demand for geriatric care increases, so does the imperative for medical education to include robust and standardized training on frailty. The global necessity for geriatric care as well as end-of-life care makes this education that much more relevant. Comprehensive understanding of frailty–including its multidimensional nature, clinical implications, and assessment strategies–is essential for future healthcare professionals. As noted by Ehrlich et al. [67], frailty is significantly associated with postoperative delirium and other acute complications, underscoring the importance of early identification and management. A more standardized and comprehensive approach to frailty, beginning with effective screening and followed by domain-specific evaluations, will better equip clinicians to deliver personalized, anticipatory care.

## Limitations

We acknowledge that our research contains limitations in that our categorization of frailty parameters into 45 distinct domains may reflect some degree of subjectivity. While we aimed to increase transparency by providing examples for each category, we acknowledge that other researchers might classify certain parameters differently. Additionally, our review did not capture the theoretical approach (deficit-accumulation, phenotypic, or combined) or the scoring methods employed by each FA. Including this information may add valuable context regarding the conceptual foundations and comparative utility of different instruments.

It is also important to contextualize our findings within the limitations of the included studies. Several reviewed articles were secondary analyses of existing datasets, in which the scope of frailty parameters was constrained by available variables. Such studies may have used proxy measures of modified versions of established FAs, which can limit conclusions about the comprehensiveness of validity of the assessments. Additionally, conclusions about geographical variation in FA use and content should be interpreted with caution, as access to full frailty domains may vary based on data availability rather than clinical preference.

We also acknowledge that this review did not apply a formal risk of bias assessment tool, which may be considered a limitation. However, this decision was based on the descriptive nature of our study, which did not evaluate outcomes or interventions based on FAs. As detailed in the Methods section, we worked to mitigate potential bias through practices such as strict inclusion/exclusion criteria, dual-review screening, and independent data extraction. Nonetheless, some degree of bias may still be present.

Another limitation relates to the categorization of composite instruments, such as the Clinical Frail Scale (CFS), which was grouped under “Other” (Category 17) due to its multidimensional nature encompassed in a single score. This categorization, while practical for our review, limits insight into the specific domains captured by CFS and other similar assessments.

Cultural and contextual differences also proved to be a challenge when analyzing our data set. Constructs such as social support, independence, or psychological distress may be understood differently across cultures, which affects both the development and application of FAs. Our review did not assess the cultural adaptability of contextual sensitivity of the analyzed instruments. As a result, some parameters may be under- or overrepresented in certain geographic contexts due to cultural norms rather than clinical reasoning.

In terms of scope, this review is not exhaustive. We limited inclusion to studies using key search terms, which may exclude some relevant literature. Furthermore, we defined “geriatric” as individuals aged 65 and older, consistent with conventions in the United States. However, age thresholds for defining older adults vary globally which may have resulted in the omission of pertinent studies from other regions. Lastly, while our review aimed to represent international FA usage, it may not capture the full spectrum of tools in use–both in the countries we included and in those not represented. Therefore, the results may not fully reflect global practices in frailty assessment.

## Further research

Further research is needed to assess the optimal frailty measures for specific health care settings. For example, the optimal parameters of frailty may differ between contexts such as perioperative medicine versus primary care. Once optimal parameters are identified, it may be beneficial to develop a standardized FA that includes parameters relevant to multiple healthcare settings. A standardized FA could then be integrated into electronic health records and used to generate medical specialty-specific frailty measures as needed. This integration of frailty assessments as a routine element of patients’ medical care could allow for frailty to be tracked over time and reveal opportunities for interventions as frailty declines. We recognize that it may be unrealistic to have one standardized FA to apply to all medical specialties as one FA may not be sufficient to capture the nuances of various specialties, but having a more standardized knowledge of what constitutes frailty may allow providers in various healthcare fields to utilize similar parameters that can allow us to track the incline/decline/stagnation of a patient’s frailty over time. This ability to monitor the dynamic nature of frailty could better inform patient care and treatment plans.

As our review only included 39 countries, it may be beneficial for additional research to be performed to gain a broader understanding of those countries not included in this study. Similarly, it would be interesting to investigate the cultural adaptability of given FAs (specifically in consideration of social and mental health parameters) and, if future researchers should choose to develop a standardized FA, how cultural differences can be addressed.

## Conclusion

With an aging population, the study and diagnosis of frailty is increasingly relevant. This analysis demonstrates that, while a wide variety of frailty assessments are utilized worldwide, frailty assessments primarily measure functional and physical health parameters when compared to mental, social, and cognitive health parameters. While research efforts continue to investigate the optimal parameters of frailty within medical settings, it is crucial that mental, social, and cognitive health parameters are included in testing as they may play an important role in frailty and adverse health outcomes.

## Financial disclosure

Research reported in this article was supported by the Institute of Aging of the National Institutes of Health under award number: 1R21AG065744-01A1

## CRediT authorship contribution statement

**Samantha Gaston:** Writing – review & editing, Writing – original draft, Methodology, Investigation, Formal analysis, Data curation, Conceptualization. **Elle Billman:** Writing – review & editing, Writing – original draft, Methodology, Investigation, Formal analysis, Conceptualization. **Lichy Han:** Writing – review & editing, Validation, Data curation. **David Drover:** Writing – review & editing, Validation, Supervision, Methodology, Conceptualization.

## Declaration of competing interest

On behalf of all authors, the corresponding author states that there is no conflict of interest.

## Appendix Supplementary materials

Image, application 1
