# Supplementary material for: Frailty assessment utilization around the globe–a systematic review
Source: J Frailty Aging. 2025 Oct 2;14(6):100088. doi: 10.1016/j.tjfa.2025.100088 (PMC12516048; doi:10.1016/j.tjfa.2025.100088)
Supplement: Supplementary file 1 [file mmc1.docx]

Supplemental Material

Articles included in systematic review.

1. Abbas M, Le Bouquin Jeannès R. Acceleration-based gait analysis for frailty assessment in older adults. Article. *Pattern Recognition Letters*. 2022;161:45-51. doi:10.1016/j.patrec.2022.07.006
2. Abbasi M, Khera S, Dabravolskaj J, et al. A cross-sectional study examining convergent validity of a frailty index based on electronic medical records in a Canadian primary care program. *BMC Geriatr*. Apr 16 2019;19(1):109. doi:10.1186/s12877-019-1119-x
3. Abete P, Basile C, Bulli G, et al. The Italian version of the "frailty index" based on deficits in health: a validation study. *Aging Clin Exp Res*. Oct 2017;29(5):913-926. doi:10.1007/s40520-017-0793-9
4. Abraham A, Burrows S, Abraham NJ, Mandal B. Modified frailty index and hypoalbuminemia as predictors of adverse outcomes in older adults presenting to acute general surgical unit. *Rev Esp Geriatr Gerontol*. Mar - Apr 2020;55(2):70-75. doi:10.1016/j.regg.2019.09.005
5. Aceto P, Perilli V, Luca E, et al. Predictive power of modified frailty index score for pulmonary complications after major abdominal surgery in the elderly: a single centre prospective cohort study. *Eur Rev Med Pharmacol Sci*. May 2021;25(10):3798-3802. doi:10.26355/eurrev_202105_25947
6. Aguilar-Frasco JL, Rodriguez-Quintero JH, Moctezuma-Velazquez P, et al. Frailty index as a predictive preoperative tool in the elder population undergoing major abdominal surgery: a prospective analysis of clinical utility. *Langenbecks Arch Surg*. Jun 2021;406(4):1189-1198. doi:10.1007/s00423-021-02128-6
7. Akti S, Zeybek H. Is the 5-factor modified Frailty Index a prognostic marker in geriatric ankle fractures? *Ulus Travma Acil Cerrahi Derg*. Mar 2022;28(3):315-319. 5 faktorlu modifiye kirilganlik indeksi, geriatrik ayak bilegi kiriklarinda prognostik bir gosterge midir? doi:10.14744/tjtes.2021.08972
8. Albakri A, Orkaby A, Rosenberg MA. Feasibility of Frailty Assessment Integrated with Cardiac Implantable Electronic Device Clinic Follow-up: A Pilot Investigation. *Gerontol Geriatr Med*. Jan-Dec 2021;7:2333721420987342. doi:10.1177/2333721420987342
9. Alegre O, Formiga F, Lopez-Palop R, et al. An Easy Assessment of Frailty at Baseline Independently Predicts Prognosis in Very Elderly Patients With Acute Coronary Syndromes. *J Am Med Dir Assoc*. Apr 2018;19(4):296-303. doi:10.1016/j.jamda.2017.10.007
10. Ali R, Schwalb JM, Nerenz DR, Antoine HJ, Rubinfeld I. Use of the modified frailty index to predict 30-day morbidity and mortality from spine surgery. *J Neurosurg Spine*. Oct 2016;25(4):537-541. doi:10.3171/2015.10.SPINE14582
11. Alqahtani BA, Abdelbasset WK, Alenazi AM. Psychometric analysis of the Arabic (Saudi) Tilburg Frailty Indicator among Saudi community-dwelling older adults. *Arch Gerontol Geriatr*. Sep - Oct 2020;90:104128. doi:10.1016/j.archger.2020.104128
12. Alqahtani BA, Nasser TA. Assessment of frailty in Saudi community-dwelling older adults: validation of measurements. *Ann Saudi Med*. May-Jun 2019;39(3):197-204. doi:10.5144/0256-4947.2019.197
13. Amblas-Novellas J, Martori JC, Molist Brunet N, Oller R, Gomez-Batiste X, Espaulella Panicot J. [Frail-VIG index: Design and evaluation of a new frailty index based on the Comprehensive Geriatric Assessment]. *Rev Esp Geriatr Gerontol*. May - Jun 2017;52(3):119-127. Indice fragil-VIG: diseno y evaluacion de un indice de fragilidad basado en la Valoracion Integral Geriatrica. doi:10.1016/j.regg.2016.09.003
14. Anand A, Cudmore S, Robertson S, et al. Frailty assessment and risk prediction by GRACE score in older patients with acute myocardial infarction. *BMC Geriatr*. Mar 13 2020;20(1):102. doi:10.1186/s12877-020-1500-9
15. Andreasen J, Lund H, Aadahl M, Gobbens RJ, Sorensen EE. Content validation of the Tilburg Frailty Indicator from the perspective of frail elderly. A qualitative explorative study. *Arch Gerontol Geriatr*. Nov-Dec 2015;61(3):392-9. doi:10.1016/j.archger.2015.08.017
16. Arosio B, Geraci A, Ferri E, Mari D, Cesari M. Biological Frailty Index in centenarians. *Aging Clin Exp Res*. Mar 2022;34(3):687-690. doi:10.1007/s40520-021-01993-x
17. Arshad MZ, Jung D, Park M, Shin H, Kim J, Mun KR. Gait-based Frailty Assessment using Image Representation of IMU Signals and Deep CNN. *Annu Int Conf IEEE Eng Med Biol Soc*. Nov 2021;2021:1874-1879. doi:10.1109/EMBC46164.2021.9630976
18. Arslan M, Meltem Koç E, Sözmen MK. The Turkish adaptation of the Tilburg Frailty Indicator: A validity and reliability study. Article. *Turk Geriatri Dergisi*. 2018;21(2):173-183. doi:10.31086/tjgeri.2018240418
19. Avvari A, Reddy BM, Ganguly E, Sharma PK. Assessment of frailty syndrome with coexisting hypertension and depression among older individuals, aged >80 years of age. *J Frailty Sarcopenia Falls*. Jun 2022;7(2):72-80. doi:10.22540/JFSF-07-072
20. Back C, Hornum M, Jorgensen MB, Lorenzen US, Olsen PS, Moller CH. Comprehensive assessment of frailty score supplements the existing cardiac surgical risk scores. *Eur J Cardiothorac Surg*. Sep 11 2021;60(3):710-716. doi:10.1093/ejcts/ezab127
21. Baldwin MR, Singer JP, Huang D, et al. Refining low physical activity measurement improves frailty assessment in lung transplant and older survivors of critical illness. Conference Abstract. *American journal of respiratory and critical care medicine Conference: american thoracic society international conference, ATS 2017 United states*. 2017;195(no pagination)doi:10.1164/ajrccm-conference.2017.A102
22. Beier F, Loffler M, Nees F, Hausner L, Frolich L, Flor H. Sensory and motor correlates of frailty: dissociation between frailty phenotype and frailty index. *BMC Geriatr*. Sep 15 2022;22(1):755. doi:10.1186/s12877-022-03416-6
23. Birkelbach O, Morgeli R, Spies C, et al. Routine frailty assessment predicts postoperative complications in elderly patients across surgical disciplines - a retrospective observational study. *BMC Anesthesiol*. Nov 7 2019;19(1):204. doi:10.1186/s12871-019-0880-x
24. Blinka MD, Buta B, Bader KD, et al. Developing a sensor-based mobile application for in-home frailty assessment: a qualitative study. *BMC Geriatr*. Feb 4 2021;21(1):101. doi:10.1186/s12877-021-02041-z
25. Boyd PJ, Nevard M, Ford JA, Khondoker M, Cross JL, Fox C. The electronic frailty index as an indicator of community healthcare service utilisation in the older population. *Age Ageing*. Mar 1 2019;48(2):273-277. doi:10.1093/ageing/afy181
26. Bras L, Peters TT, Wedman J, et al. Predictive value of the Groningen Frailty Indicator for treatment outcomes in elderly patients after head and neck, or skin cancer surgery in a retrospective cohort. *Clin Otolaryngol*. Oct 2015;40(5):474-82. doi:10.1111/coa.12409
27. Braun T, Gruneberg C, Thiel C. German translation, cross-cultural adaptation and diagnostic test accuracy of three frailty screening tools : PRISMA-7, FRAIL scale and Groningen Frailty Indicator. *Z Gerontol Geriatr*. Apr 2018;51(3):282-292. Deutsche Ubersetzung, interkulturelle Adaptation und diagnostische Testgenauigkeit von drei Frailty Screening Fragebogen : PRISMA-7, FRAIL Skala und Groningen Frailty Indicator. doi:10.1007/s00391-017-1295-2
28. Breccia M, Palandri F, Luciano L, et al. Identification and assessment of frailty in older patients with chronic myeloid leukemia and myelofibrosis, and indications for tyrosine kinase inhibitor treatment. *Ann Hematol*. May 2018;97(5):745-754. doi:10.1007/s00277-018-3258-0
29. Broad A, Carter B, McKelvie S, Hewitt J. The Convergent Validity of the electronic Frailty Index (eFI) with the Clinical Frailty Scale (CFS). *Geriatrics (Basel)*. Nov 9 2020;5(4)doi:10.3390/geriatrics5040088
30. Brousseau AA, Dent E, Hubbard R, et al. Identification of older adults with frailty in the Emergency Department using a frailty index: results from a multinational study. *Age Ageing*. Mar 1 2018;47(2):242-248. doi:10.1093/ageing/afx168
31. Brown JD, Alipour-Haris G, Pahor M, Manini TM. Association between a Deficit Accumulation Frailty Index and Mobility Outcomes in Older Adults: Secondary Analysis of the Lifestyle Interventions and Independence for Elders (LIFE) Study. *J Clin Med*. Nov 22 2020;9(11)doi:10.3390/jcm9113757
32. Brundle C, Heaven A, Brown L, et al. Convergent validity of the electronic frailty index. *Age Ageing*. Jan 1 2019;48(1):152-156. doi:10.1093/ageing/afy162
33. Burn R, Hubbard RE, Scrase RJ, et al. A frailty index derived from a standardized comprehensive geriatric assessment predicts mortality and aged residential care admission. *BMC Geriatr*. Dec 27 2018;18(1):319. doi:10.1186/s12877-018-1016-8
34. Butkuviene M, Tamuleviciute-Prasciene E, Beigiene A, et al. Wearable-Based Assessment of Frailty Trajectories During Cardiac Rehabilitation After Open-Heart Surgery. *IEEE J Biomed Health Inform*. Sep 2022;26(9):4426-4435. doi:10.1109/JBHI.2022.3181738
35. Çakmak G, Öztürk ZA. The relationship between polypharmacy and frailty in older adults: Which frailty assessment tool shows the relationship best? Article. *Progress in Nutrition*. 2021;23(3)e2021295. doi:10.23751/pn.v23i3.12036
36. Callahan KE, Clark CJ, Edwards AF, et al. Automated Frailty Screening At-Scale for Pre-Operative Risk Stratification Using the Electronic Frailty Index. *J Am Geriatr Soc*. May 2021;69(5):1357-1362. doi:10.1111/jgs.17027
37. Carter B, Keevil VL, Anand A, et al. The Prognostic and Discriminatory Utility of the Clinical Frailty Scale and Modified Frailty Index Compared to Age. *Geriatrics (Basel)*. Aug 24 2022;7(5)doi:10.3390/geriatrics7050087
38. Cella A, Ferrari A, Rengo G, et al. Agreement of a Short Form of the Self-Administered Multidimensional Prognostic Index (SELFY-MPI-SF): A Useful Tool for the Self-Assessment of Frailty in Community-Dwelling Older People. *Clin Interv Aging*. 2020;15:493-499. doi:10.2147/CIA.S241721
39. Cesari M, Franchi C, Cortesi L, et al. Implementation of the Frailty Index in hospitalized older patients: Results from the REPOSI register. *Eur J Intern Med*. Oct 2018;56:11-18. doi:10.1016/j.ejim.2018.06.001
40. Checa-Lopez M, Oviedo-Briones M, Pardo-Gomez A, et al. FRAILTOOLS study protocol: a comprehensive validation of frailty assessment tools to screen and diagnose frailty in different clinical and social settings and to provide instruments for integrated care in older adults. *BMC Geriatr*. Mar 18 2019;19(1):86. doi:10.1186/s12877-019-1042-1
41. Chen Q, Tang B, Zhai Y, et al. Dynamic statistical model for predicting the risk of death among older Chinese people, using longitudinal repeated measures of the frailty index: a prospective cohort study. *Age Ageing*. Oct 23 2020;49(6):966-973. doi:10.1093/ageing/afaa056
42. Chen Y, Qin J. Modified Frailty Index Independently Predicts Postoperative Delirium and Delayed Neurocognitive Recovery After Elective Total Joint Arthroplasty. *J Arthroplasty*. Feb 2021;36(2):449-453. doi:10.1016/j.arth.2020.07.074
43. Cheng D, Dumontier C, Sheikh AR, et al. Prognostic value of the veterans affairs frailty index in older patients with non-small cell lung cancer. *Cancer Med*. Aug 2022;11(15):3009-3022. doi:10.1002/cam4.4658
44. Cheng D, DuMontier C, Yildirim C, et al. Updating and Validating the U.S. Veterans Affairs Frailty Index: Transitioning From ICD-9 to ICD-10. *J Gerontol A Biol Sci Med Sci*. Jun 14 2021;76(7):1318-1325. doi:10.1093/gerona/glab071
45. Chkeir A, Safieddine D, Chehade F, et al. Is there a relationship between frailty indices and balance assessment in older people? 2016:
46. Choi J, Marafino BJ, Vendrow EB, et al. Rib Fracture Frailty Index: A risk stratification tool for geriatric patients with multiple rib fractures. *J Trauma Acute Care Surg*. Dec 1 2021;91(6):932-939. doi:10.1097/TA.0000000000003390
47. Clegg A, Bates C, Young J, et al. Development and validation of an electronic frailty index using routine primary care electronic health record data. *Age Ageing*. May 2016;45(3):353-60. doi:10.1093/ageing/afw039
48. Coelho T, Santos R, Paul C, Gobbens RJ, Fernandes L. Portuguese version of the Tilburg Frailty Indicator: Transcultural adaptation and psychometric validation. *Geriatr Gerontol Int*. Aug 2015;15(8):951-60. doi:10.1111/ggi.12373
49. Costa G, Bersigotti L, Massa G, et al. The Emergency Surgery Frailty Index (EmSFI): development and internal validation of a novel simple bedside risk score for elderly patients undergoing emergency surgery. *Aging Clin Exp Res*. Aug 2021;33(8):2191-2201. doi:10.1007/s40520-020-01735-5
50. Covino M, Russo A, Salini S, et al. Frailty Assessment in the Emergency Department for Risk Stratification of COVID-19 Patients Aged ≥80 Years. Article. *Journal of the American Medical Directors Association*. 2021;22(9):1845-1852.e1. doi:10.1016/j.jamda.2021.07.005
51. Covino M, Salini S, Russo A, et al. Frailty Assessment in the Emergency Department for Patients >/=80 Years Undergoing Urgent Major Surgical Procedures. *J Am Med Dir Assoc*. Apr 2022;23(4):581-588. doi:10.1016/j.jamda.2021.12.039
52. Cramer CL, Kane WJ, Lattimore CM, Turrentine FE, Zaydfudim VM. Evaluating the Impact of Preoperative Geriatric-Specific Variables and Modified Frailty Index on Postoperative Outcomes After Elective Pancreatic Surgery. *World J Surg*. Sep 8 2022;doi:10.1007/s00268-022-06710-x
53. Crozier-Shaw G, Joyce WP. Too frail for surgery? A frailty index in major colorectal surgery. *ANZ J Surg*. Dec 2018;88(12):1302-1305. doi:10.1111/ans.14792
54. Cullinan S, O'Mahony D, O'Sullivan D, Byrne S. Use of a frailty index to identify potentially inappropriate prescribing and adverse drug reaction risks in older patients. *Age Ageing*. Jan 2016;45(1):115-20. doi:10.1093/ageing/afv166
55. D'Cruz R T, Chong TT, Tan TF, et al. The Modified Frailty Index Does Not Predict Mortality After Major Lower Extremity Amputation for Peripheral Arterial Disease in an Asian Population. *Ann Vasc Surg*. Nov 2020;69:298-306. doi:10.1016/j.avsg.2020.05.063
56. Dai M, Yue J, Zhang J, Wang H, Wu C. Functional dentition is a modifier of the association between vitamin D and the frailty index among Chinese older adults: a population-based longitudinal study. *BMC Geriatr*. Feb 28 2022;22(1):159. doi:10.1186/s12877-022-02857-3
57. Dallmeier D, Braisch U, Rapp K, et al. Frailty Index and Sex-Specific 6-Year Mortality in Community-Dwelling Older People: The ActiFE Study. *J Gerontol A Biol Sci Med Sci*. Jan 20 2020;75(2):366-373. doi:10.1093/gerona/glz051
58. Dammeyer K, Alfonso AR, Diep GK, et al. Predicting postoperative complications following mastectomy in the elderly: Evidence for the 5-factor frailty index. *Breast J*. Jun 2021;27(6):509-513. doi:10.1111/tbj.14208
59. De Nunzio C, Cicione A, Izquierdo L, et al. Multicenter Analysis of Postoperative Complications in Octogenarians After Radical Cystectomy and Ureterocutaneostomy: The Role of the Frailty Index. *Clin Genitourin Cancer*. Oct 2019;17(5):402-407. doi:10.1016/j.clgc.2019.07.002
60. Duzgun G, Ustundag S, Karadakovan A. Assessment of Frailty in the Elderly. *Florence Nightingale J Nurs*. Feb 2021;29(1):2-8. doi:10.5152/FNJN.2021.414736
61. Eoh KJ, Yoon JW, Lee JY, et al. A novel algorithm for the treatment strategy for advanced epithelial ovarian cancer: consecutive imaging, frailty assessment, and diagnostic laparoscopy. *BMC Cancer*. Jul 12 2017;17(1):481. doi:10.1186/s12885-017-3476-1
62. Espaulella-Ferrer M, Espaulella-Panicot J, Noell-Boix R, et al. Assessment of frailty in elderly patients attending a multidisciplinary wound care centre: a cohort study. *BMC Geriatr*. Dec 18 2021;21(1):727. doi:10.1186/s12877-021-02676-y
63. Espinoza SE, Woods RL, Ekram A, et al. The effect of low-dose aspirin on frailty phenotype and frailty index in community-dwelling older adults in the ASPirin in Reducing Events in the Elderly study. Journal article. *Journals of gerontology Series A, Biological sciences and medical sciences*. 2021;doi:10.1093/gerona/glab340
64. Esses G, Andreopoulos E, Lin HM, Arya S, Deiner S. A Comparison of Three Frailty Indices in Predicting Morbidity and Mortality After On-Pump Aortic Valve Replacement. *Anesth Analg*. Jan 2018;126(1):39-45. doi:10.1213/ANE.0000000000002411
65. Fallon A, Kilbane L, Briggs R, et al. Screening for frailty in older emergency department patients: the utility of the Survey of Health, Ageing and Retirement in Europe Frailty Instrument. *QJM*. Mar 1 2018;111(3):151-154. doi:10.1093/qjmed/hcx242
66. Federico A, Caprio GG, Dalise AM, et al. Cirrhosis and frailty assessment in elderly patients: A paradoxical result. *Medicine (Baltimore)*. Jan 2020;99(2):e18501. doi:10.1097/MD.0000000000018501
67. Feenstra M, Oud FMM, Jansen CJ, Smidt N, van Munster BC, de Rooij SE. Reproducibility and responsiveness of the Frailty Index and Frailty Phenotype in older hospitalized patients. *BMC Geriatr*. Sep 17 2021;21(1):499. doi:10.1186/s12877-021-02444-y
68. Fougere B, Kelaiditi E, Hoogendijk EO, et al. Frailty Index and Quality of Life in Nursing Home Residents: Results From INCUR Study. *J Gerontol A Biol Sci Med Sci*. Mar 2016;71(3):420-4. doi:10.1093/gerona/glv098
69. Fujisawa C, Umegaki H, Sugimoto T, et al. Older adults with a higher frailty index tend to have electrolyte imbalances. *Exp Gerontol*. Jun 15 2022;163:111778. doi:10.1016/j.exger.2022.111778
70. Ga H, Won CW, Jung HW. Use of the Frailty Index and FRAIL-NH Scale for the Assessment of the Frailty Status of Elderly Individuals Admitted in a Long-term Care Hospital in Korea. *Ann Geriatr Med Res*. Mar 2018;22(1):20-25. doi:10.4235/agmr.2018.22.1.20
71. Garcia-Garcia FJ, Carnicero JA, Losa-Reyna J, et al. Frailty Trait Scale-Short Form: A Frailty Instrument for Clinical Practice. *J Am Med Dir Assoc*. Sep 2020;21(9):1260-1266 e2. doi:10.1016/j.jamda.2019.12.008
72. Garcia-Moreno FM, Bermudez-Edo M, Garrido JL, Rodriguez-Garcia E, Perez-Marmol JM, Rodriguez-Fortiz MJ. A Microservices e-Health System for Ecological Frailty Assessment Using Wearables. *Sensors (Basel)*. Jun 17 2020;20(12)doi:10.3390/s20123427
73. García-Pérez E, Aguirre-Larracoechea U, Portugal-Porras V, Azpiazu-Landa N, Telletxea-Benguria S. Frailty assessment has come to stay: Retrospective analysis pilot study of two frailty scales in oncological older patients undergoing colorectal surgery. Article. *Revista Espanola de Anestesiologia y Reanimacion*. 2022;doi:10.1016/j.redar.2021.05.028
74. Geessink NH, Schoon Y, Olde Rikkert MG, Melis RJ. Criterion Validity of a Frailty Index Derived from the Easycare Instrument. *J Am Geriatr Soc*. Jan 2017;65(1):222-224. doi:10.1111/jgs.14546
75. Giaccherini L, Galaverni M, Renna I, et al. Role of multidimensional assessment of frailty in predicting outcomes in older patients with glioblastoma treated with adjuvant concurrent chemo-radiation. *J Geriatr Oncol*. Sep 2019;10(5):770-778. doi:10.1016/j.jgo.2019.03.009
76. Giannotti C, Sambuceti S, Signori A, et al. Frailty assessment in elective gastrointestinal oncogeriatric surgery: Predictors of one-year mortality and functional status. *J Geriatr Oncol*. Sep 2019;10(5):716-723. doi:10.1016/j.jgo.2019.04.017
77. Giroux M, Sirois MJ, Boucher V, et al. Frailty Assessment to Help Predict Patients at Risk of Delirium When Consulting the Emergency Department. *J Emerg Med*. Aug 2018;55(2):157-164. doi:10.1016/j.jemermed.2018.02.032
78. Gobbens RJJ, Andreasen J. The prediction of readmission and mortality by the domains and components of the Tilburg Frailty Indicator (TFI): A prospective cohort study among acutely admitted older patients. *Arch Gerontol Geriatr*. Jul - Aug 2020;89:104077. doi:10.1016/j.archger.2020.104077
79. Gobbens RJJ, Andreasen J. Multidimensional frailty and its determinants among acutely admitted older people: a cross-sectional study using the Tilburg Frailty Indicator. *Eur Geriatr Med*. Feb 2021;12(1):151-160. doi:10.1007/s41999-020-00388-x
80. Gobbens RJJ, Boersma P, Uchmanowicz I, Santiago LM. The tilburg frailty indicator (TFI): New evidence for its validity. Article. *Clinical Interventions in Aging*. 2020;15:265-274. doi:10.2147/CIA.S243233
81. Gobbens RJJ, van Assen M, Augustijn H, Goumans M, van der Ploeg T. Prediction of Mortality by the Tilburg Frailty Indicator (TFI). *J Am Med Dir Assoc*. Mar 2021;22(3):607 e1-607 e6. doi:10.1016/j.jamda.2020.07.033
82. Gobbens RJJ, van der Ploeg T. The Development of Multidimensional Frailty Over Seven Years A longitudinal study among Dutch community-dwelling older people using the Tilburg Frailty Indicator. *Arch Gerontol Geriatr*. Jul-Aug 2021;95:104393. doi:10.1016/j.archger.2021.104393
83. Goldstein J, Hubbard RE, Moorhouse P, Andrew MK, Mitnitski A, Rockwood K. The validation of a care partner-derived frailty index based upon comprehensive geriatric assessment (CP-FI-CGA) in emergency medical services and geriatric ambulatory care. *Age Ageing*. Mar 2015;44(2):327-30. doi:10.1093/ageing/afu161
84. Gu C, Lu A, Lei C, et al. Frailty index is useful for predicting postoperative morbidity in older patients undergoing gastrointestinal surgery: a prospective cohort study. *BMC Surg*. Feb 16 2022;22(1):57. doi:10.1186/s12893-022-01471-9
85. Guan L, Soh CH, Reijnierse EM, Lim WK, Maier AB. Association of a modified laboratory frailty index with adverse outcomes in geriatric rehabilitation inpatients: RESORT. *Mech Ageing Dev*. Apr 2022;203:111648. doi:10.1016/j.mad.2022.111648
86. Guerard EJ, Deal AM, Chang Y, et al. Frailty Index Developed From a Cancer-Specific Geriatric Assessment and the Association With Mortality Among Older Adults With Cancer. *J Natl Compr Canc Netw*. Jul 2017;15(7):894-902. doi:10.6004/jnccn.2017.0122
87. Guiab K, Evans T, Siddiqi M, et al. Can the 5-item Modified Frailty Index Predict Outcomes in Geriatric Trauma? A National Database Study. *World J Surg*. Oct 2022;46(10):2328-2334. doi:10.1007/s00268-022-06637-3
88. Hansen TK, Damsgaard EM, Shahla S, Bruun JM, Gregersen M. A reliable and record-based frailty assessment method for older medical inpatients. *Eur Geriatr Med*. Oct 2020;11(5):803-812. doi:10.1007/s41999-020-00345-8
89. Hao Q, Sun X, Yang M, Dong B, Dong B, Wei Y. Prediction of mortality in Chinese very old people through the frailty index based on routine laboratory data. *Sci Rep*. Jan 18 2019;9(1):221. doi:10.1038/s41598-018-36569-9
90. Hii TBK, Lainchbury JG, Bridgman PG. Frailty in Acute Cardiology: Comparison of a Quick Clinical Assessment Against a Validated Frailty Assessment Tool. Article. *Heart Lung and Circulation*. 2015;24(6):551-556. doi:10.1016/j.hlc.2014.11.024
91. Hollinghurst J, Fry R, Akbari A, et al. External validation of the electronic Frailty Index using the population of Wales within the Secure Anonymised Information Linkage Databank. *Age Ageing*. Nov 1 2019;48(6):922-926. doi:10.1093/ageing/afz110
92. Hoogendijk EO, Abellan van Kan G, Guyonnet S, Vellas B, Cesari M. Components of the Frailty Phenotype in Relation to the Frailty Index: Results From the Toulouse Frailty Platform. Article. *Journal of the American Medical Directors Association*. 2015;16(10):855-859. doi:10.1016/j.jamda.2015.04.007
93. Hoogendijk EO, Stenholm S, Ferrucci L, Bandinelli S, Inzitari M, Cesari M. Operationalization of a frailty index among older adults in the InCHIANTI study: predictive ability for all-cause and cardiovascular disease mortality. *Aging Clin Exp Res*. Jun 2020;32(6):1025-1034. doi:10.1007/s40520-020-01478-3
94. Hoogendijk EO, van Kan GA, Guyonnet S, Vellas B, Cesari M. Components of the Frailty Phenotype in Relation to the Frailty Index: Results From the Toulouse Frailty Platform. *J Am Med Dir Assoc*. Oct 1 2015;16(10):855-9. doi:10.1016/j.jamda.2015.04.007
95. Hosler QP, Maltagliati AJ, Shi SM, et al. A Practical Two-Stage Frailty Assessment for Older Adults Undergoing Aortic Valve Replacement. *J Am Geriatr Soc*. Oct 2019;67(10):2031-2037. doi:10.1111/jgs.16036
96. Hubbard RE, Peel NM, Samanta M, et al. Derivation of a frailty index from the interRAI acute care instrument. *BMC Geriatr*. Mar 18 2015;15:27. doi:10.1186/s12877-015-0026-z
97. Hung CD, Yang CC, Lee CY, et al. Polypharmacy is significantly and positively associated with the frailty status assessed using the 5-item frail scale, cardiovascular health phenotypic classification of frailty index, and study of osteoporotic fractures scale. Article. *Journal of Clinical Medicine*. 2021;10(19)4413. doi:10.3390/jcm10194413
98. Jager J, Sieber CC, Gassmann KG, Ritt M. Changes of a frailty index based on common blood and urine tests during a hospital stay on geriatric wards predict 6-month and 1-year mortality in older people. *Clin Interv Aging*. 2019;14:473-484. doi:10.2147/CIA.S191117
99. Jakobsson U. Measuring Frailty among Older People: Further Evaluation of the Brody Frailty Index. *J Frailty Aging*. 2017;6(4):224-227. doi:10.14283/jfa.2017.41
100. Jarman H, Crouch R, Baxter M, Cole E, Dillane B, Wang C. Frailty in major trauma study (FRAIL-T): a study protocol to determine the feasibility of nurse-led frailty assessment in elderly trauma and the impact on outcome in patients with major trauma. *BMJ Open*. Aug 5 2020;10(8):e038082. doi:10.1136/bmjopen-2020-038082
101. Ji S, Baek JY, Jin T, Lee E, Jang IY, Jung HW. Association Between Changes in Frailty Index and Clinical Outcomes: An Observational Cohort Study. *Clin Interv Aging*. 2022;17:627-636. doi:10.2147/CIA.S358512
102. Ju C, Zhou J, Lee S, et al. Derivation of an electronic frailty index for predicting short-term mortality in heart failure: a machine learning approach. *ESC Heart Fail*. Aug 2021;8(4):2837-2845. doi:10.1002/ehf2.13358
103. Jung D, Kim J, Kim M, Won CW, Mun KR. Frailty Assessment Using Temporal Gait Characteristics and a Long Short-Term Memory Network. *IEEE J Biomed Health Inform*. Sep 2021;25(9):3649-3658. doi:10.1109/JBHI.2021.3067931
104. Jung HW, Baek JY, Jang IY, et al. Short Physical Performance Battery as a Crosswalk Between Frailty Phenotype and Deficit Accumulation Frailty Index. *J Gerontol A Biol Sci Med Sci*. Nov 15 2021;76(12):2249-2255. doi:10.1093/gerona/glab087
105. Jung HW, Kim S, Won CW. Validation of the Korean Frailty Index in community-dwelling older adults in a nationwide Korean Frailty and Aging Cohort study. *Korean J Intern Med*. Mar 2021;36(2):456-466. doi:10.3904/kjim.2019.172
106. Kameyama H, Sakata J, Hanyu T, et al. Efficacy of preoperative frailty assessment in patients with gastrointestinal disease. *Geriatr Gerontol Int*. Mar 2021;21(3):327-330. doi:10.1111/ggi.14134
107. Kang GE, Naik AD, Ghanta RK, Rosengart TK, Najafi B. A Wrist-Worn Sensor-Derived Frailty Index Based on an Upper-Extremity Functional Test in Predicting Functional Mobility in Older Adults. *Gerontology*. 2021;67(6):753-761. doi:10.1159/000515078
108. Kapadia M, Obaid O, Nelson A, et al. Evaluation of Frailty Assessment Compliance in Acute Care Surgery: Changing Trends, Lessons Learned. *J Surg Res*. Feb 2022;270:236-244. doi:10.1016/j.jss.2021.09.019
109. Kelaiditi E, Andrieu S, Cantet C, Vellas B, Cesari M, Group ID. Frailty Index and Incident Mortality, Hospitalization, and Institutionalization in Alzheimer's Disease: Data From the ICTUS Study. *J Gerontol A Biol Sci Med Sci*. Apr 2016;71(4):543-8. doi:10.1093/gerona/glv137
110. Kelaiditi E, Canevelli M, Andrieu S, et al. Frailty Index and Cognitive Decline in Alzheimer's Disease: Data from the Impact of Cholinergic Treatment USe Study. *J Am Geriatr Soc*. Jun 2016;64(6):1165-70. doi:10.1111/jgs.13956
111. Kerminen H, Huhtala H, Jantti P, Valvanne J, Jamsen E. Frailty Index and functional level upon admission predict hospital outcomes: an interRAI-based cohort study of older patients in post-acute care hospitals. *BMC Geriatr*. May 5 2020;20(1):160. doi:10.1186/s12877-020-01550-7
112. Kesserwan M, Bergin B, Trivedi A, et al. Assessment of Frailty in Predicting Surgical Outcomes in Patients with Chronic Subdural Hematomas: Retrospective Chart Review. *World Neurosurg*. Feb 2021;146:e168-e174. doi:10.1016/j.wneu.2020.10.061
113. Khadka J, Visvanathan R, Theou O, et al. Development and validation of a frailty index based on Australian Aged Care Assessment Program data. *Med J Aust*. Oct 2020;213(7):321-326. doi:10.5694/mja2.50720
114. Khamis R, Sabbah H, Sabbah S, Peters L, Droubi N, Sabbah I. Evaluating the psychometric properties of the Arabic version of the Groningen Frailty Indicator among Lebanese elderly people. *J Egypt Public Health Assoc*. Dec 23 2019;94(1):28. doi:10.1186/s42506-019-0028-3
115. Kiani S, Stebbins A, Thourani VH, et al. The Effect and Relationship of Frailty Indices on Survival After Transcatheter Aortic Valve Replacement. Article. *JACC: Cardiovascular Interventions*. 2020;13(2):219-231. doi:10.1016/j.jcin.2019.08.015
116. Kim DH, Glynn RJ, Avorn J, et al. Validation of a Claims-Based Frailty Index Against Physical Performance and Adverse Health Outcomes in the Health and Retirement Study. *J Gerontol A Biol Sci Med Sci*. Jul 12 2019;74(8):1271-1276. doi:10.1093/gerona/gly197
117. Kim DH, Schneeweiss S, Glynn RJ, Lipsitz LA, Rockwood K, Avorn J. Measuring Frailty in Medicare Data: Development and Validation of a Claims-Based Frailty Index. *J Gerontol A Biol Sci Med Sci*. Jun 14 2018;73(7):980-987. doi:10.1093/gerona/glx229
118. Kim JY, Park IS, Kang DH, Lee YS, Kim KT, Hong SJ. Prediction of risk factors after spine surgery in patients aged &gt;75 years using the modified frailty index. Article. *Journal of Korean Neurosurgical Society*. 2020;63(6):827-833. doi:10.3340/jkns.2020.0019
119. Kim SJ, Fessele KL, Tin AL, et al. The association between Memorial Sloan Kettering Frailty Index with 30-day survival among patients aged ≥ 75 with cancer and COVID-19. Article. *Journal of Geriatric Oncology*. 2022;13(4):416-419. doi:10.1016/j.jgo.2021.12.014
120. Kim SW, Yoon SJ, Choi JY, et al. Clinical implication of frailty assessment in older patients with atrial fibrillation. *Arch Gerontol Geriatr*. May - Jun 2017;70:1-7. doi:10.1016/j.archger.2016.12.001
121. Kim Y, Song K, Kang CM, Lee H. Impact of preoperative laboratory frailty index on mortality and clinical outcomes in older surgical patients with cancer. *Sci Rep*. Jun 2 2022;12(1):9200. doi:10.1038/s41598-022-13426-4
122. King KE, Fillenbaum GG, Cohen HJ. A Cumulative Deficit Laboratory Test–based Frailty Index: Personal and Neighborhood Associations. Article. *Journal of the American Geriatrics Society*. 2017;65(9):1981-1987. doi:10.1111/jgs.14983
123. Kinosian B, Wieland D, Gu X, Stallard E, Phibbs CS, Intrator O. Validation of the JEN frailty index in the National Long-Term Care Survey community population: identifying functionally impaired older adults from claims data. *BMC Health Serv Res*. Nov 29 2018;18(1):908. doi:10.1186/s12913-018-3689-2
124. Knight T, Atkin C, Martin FC, et al. Frailty assessment and acute frailty service provision in the UK: results of a national 'day of care' survey. *BMC Geriatr*. Jan 3 2022;22(1):19. doi:10.1186/s12877-021-02679-9
125. Kojima G, Taniguchi Y, Kitamura A, Shinkai S. Are the Kihon Checklist and the Kaigo-Yobo Checklist Compatible With the Frailty Index? *J Am Med Dir Assoc*. Sep 2018;19(9):797-800 e2. doi:10.1016/j.jamda.2018.05.012
126. Kubala M, Guedon-Moreau L, Anselme F, et al. Utility of Frailty Assessment for Elderly Patients Undergoing Cardiac Resynchronization Therapy. *JACC Clin Electrophysiol*. Dec 26 2017;3(13):1523-1533. doi:10.1016/j.jacep.2017.06.012
127. Kundi H, Coskun N, Yesiltepe M. Association of entirely claims-based frailty indices with long-term outcomes in patients with acute myocardial infarction, heart failure, or pneumonia: a nationwide cohort study in Turkey. *Lancet Reg Health Eur*. Nov 2021;10:100183. doi:10.1016/j.lanepe.2021.100183
128. Kweh B, Lee H, Tan T, et al. Spinal Surgery in Patients Aged 80 Years and Older: Risk Stratification Using the Modified Frailty Index. *Global Spine J*. May 2021;11(4):525-532. doi:10.1177/2192568220914877
129. Kweh BTS, Lee HQ, Tan T, Liew S, Hunn M, Wee Tee J. Posterior Instrumented Spinal Surgery Outcomes in the Elderly: A Comparison of the 5-Item and 11-Item Modified Frailty Indices. *Global Spine J*. Aug 15 2022:21925682221117139. doi:10.1177/21925682221117139
130. Kweh BTS, Lee HQ, Tan T, et al. Risk Stratification of Elderly Patients Undergoing Spinal Surgery Using the Modified Frailty Index. *Global Spine J*. Mar 22 2021:2192568221999650. doi:10.1177/2192568221999650
131. Kwon M, Kim SA, Roh JL, et al. An Introduction to a Head and Neck Cancer-Specific Frailty Index and Its Clinical Implications in Elderly Patients: A Prospective Observational Study Focusing on Respiratory and Swallowing Functions. *Oncologist*. Sep 2016;21(9):1091-8. doi:10.1634/theoncologist.2016-0008
132. Lai HY, Huang ST, Chen LK, Hsiao FY. Development of frailty index using ICD-10 codes to predict mortality and rehospitalization of older adults: An update of the multimorbidity frailty index. *Arch Gerontol Geriatr*. May-Jun 2022;100:104646. doi:10.1016/j.archger.2022.104646
133. Lam FMH, Leung JCS, Kwok TCY. The Clinical Potential of Frailty Indicators on Identifying Recurrent Fallers in the Community: The Mr. Os and Ms. OS Cohort Study in Hong Kong. Article. *Journal of the American Medical Directors Association*. 2019;20(12):1605-1610. doi:10.1016/j.jamda.2019.06.019
134. Lansbury LN, Roberts HC, Clift E, Herklots A, Robinson N, Sayer AA. Use of the electronic Frailty Index to identify vulnerable patients: a pilot study in primary care. *Br J Gen Pract*. Nov 2017;67(664):e751-e756. doi:10.3399/bjgp17X693089
135. Larsen RT, Turcotte LA, Westendorp R, Langberg H, Hirdes JP. Frailty Index Status of Canadian Home Care Clients Improves With Exercise Therapy and Declines in the Presence of Polypharmacy. *J Am Med Dir Assoc*. Jun 2020;21(6):766-771 e1. doi:10.1016/j.jamda.2020.01.004
136. Lascano D, Pak JS, Kates M, et al. Validation of a frailty index in patients undergoing curative surgery for urologic malignancy and comparison with other risk stratification tools. *Urol Oncol*. Oct 2015;33(10):426 e1-12. doi:10.1016/j.urolonc.2015.06.002
137. Laukli I, Sandvik L, Ormstad H. Frailty assessment of older adults, first-time applicants of public home care service in Norway. *Scand J Prim Health Care*. Mar 2021;39(1):3-9. doi:10.1080/02813432.2021.1880069
138. Lee H, Tan C, Tran V, et al. The Utility of the Modified Frailty Index in Outcome Prediction for Elderly Patients with Acute Traumatic Subdural Hematoma. *J Neurotrauma*. Dec 1 2020;37(23):2499-2506. doi:10.1089/neu.2019.6943
139. Lee J, Alfonso AR, Kantar RS, et al. Modified Frailty Index Predicts Postoperative Complications following Panniculectomy in the Elderly. *Plast Reconstr Surg Glob Open*. Jul 2020;8(7):e2987. doi:10.1097/GOX.0000000000002987
140. Li L, Li H, He L, Chen H, Li Y. Study on the Relationship Between Orthostatic Hypotension and Heart Rate Variability, Pulse Wave Velocity Index, and Frailty Index in the Elderly: A Retrospective Observational Study. *Front Cardiovasc Med*. 2020;7:603957. doi:10.3389/fcvm.2020.603957
141. Li YX, Jiang XY, Stone C, et al. A new physical-cognitive scale for assessment of frailty in Chinese Han elderly. *Neurol Res*. Aug 2019;41(8):728-733. doi:10.1080/01616412.2019.1609164
142. Liang YD, Xie YB, Du MH, Shi J, Yang JF, Wang H. Development and Validation of an Electronic Frailty Index Using Routine Electronic Health Records: An Observational Study From a General Hospital in China. *Front Med (Lausanne)*. 2021;8:731445. doi:10.3389/fmed.2021.731445
143. Lim S, Jacques F, Babaki S, et al. Preoperative physical frailty assessment among octogenarians undergoing cardiac surgery: Upgrading the "eyeball" test. *J Thorac Cardiovasc Surg*. Apr 9 2021;doi:10.1016/j.jtcvs.2021.02.100
144. Lin CH, Liu CY, Rong JR. Psychometric Properties of the Taiwanese Version of the Tilburg Frailty Indicator for Community-Dwelling Older Adults. *Healthcare (Basel)*. Sep 10 2021;9(9)doi:10.3390/healthcare9091193
145. Lin H, Peel NM, Scott IA, et al. Perioperative assessment of older surgical patients using a frailty index-feasibility and association with adverse post-operative outcomes. *Anaesth Intensive Care*. Nov 2017;45(6):676-682. doi:10.1177/0310057X1704500605
146. Lin HR, Tsuji T, Kondo K, Imanaka Y. Development of a risk score for the prediction of incident dementia in older adults using a frailty index and health checkup data: The JAGES longitudinal study. *Prev Med*. Jul 2018;112:88-96. doi:10.1016/j.ypmed.2018.04.004
147. Liu W, Zhang L, Fang H, et al. Genetically predicted frailty index and risk of stroke and Alzheimer's disease. *Eur J Neurol*. Jul 2022;29(7):1913-1921. doi:10.1111/ene.15332
148. Liu Y, Kate MacPhedran A, Luo Y. Statistical measurement and analysis on how the Late-Life Function &amp; Disability Instrument enhances the frailty assessment compared to the national standards used on transcatheter aortic valve patients. 2016:433-440.
149. Liu Z, Wang Q, Zhi T, et al. Frailty Index and Its Relation to Falls and Overnight Hospitalizations in Elderly Chinese People: A Population-based Study. *J Nutr Health Aging*. 2016;20(5):561-8. doi:10.1007/s12603-015-0625-6
150. Lo SY, Zhang M, Hubbard RE, Gnjidic D, Redston MR, Hilmer SN. Development and validation of a frailty index based on data routinely collected across multiple domains in NSW hospitals. *Australas J Ageing*. Jun 2021;40(2):184-194. doi:10.1111/ajag.12888
151. Lu J, Zheng HL, Li P, et al. High preoperative modified frailty index has a negative impact on short- and long-term outcomes of octogenarians with gastric cancer after laparoscopic gastrectomy. *Surg Endosc*. May 2018;32(5):2193-2200. doi:10.1007/s00464-018-6085-4
152. Ludwig C, Busnel C. Derivation of a frailty index from the resident assessment instrument - home care adapted for Switzerland: a study based on retrospective data analysis. *BMC Geriatr*. Sep 7 2017;17(1):205. doi:10.1186/s12877-017-0604-3
153. Lv S, Ling L, Shi H, et al. Application of Muscle Thickness and Quality Measured by Ultrasound in Frailty Assessment in China. *Front Med (Lausanne)*. 2022;9:859555. doi:10.3389/fmed.2022.859555
154. Ma T, Cai J, Zhu YS, et al. Association between a frailty index based on common laboratory tests and QTc prolongation in older adults: the Rugao Longevity and Ageing Study. *Clin Interv Aging*. 2018;13:797-804. doi:10.2147/CIA.S149791
155. MacKenzie HT, Tugwell B, Rockwood K, Theou O. Frailty and Diabetes in Older Hospitalized Adults: The Case for Routine Frailty Assessment. *Can J Diabetes*. Apr 2020;44(3):241-245 e1. doi:10.1016/j.jcjd.2019.07.001
156. Madan SA, Fida N, Barman P, et al. Frailty Assessment in Advanced Heart Failure. *J Card Fail*. Oct 2016;22(10):840-4. doi:10.1016/j.cardfail.2016.02.003
157. Mah SJ, Anpalagan T, Marcucci M, et al. The five-factor modified frailty index predicts adverse postoperative and chemotherapy outcomes in gynecologic oncology. *Gynecol Oncol*. Jul 2022;166(1):154-161. doi:10.1016/j.ygyno.2022.05.012
158. Marshall L, Griffin R, Mundy J. Frailty assessment to predict short term outcomes after cardiac surgery. *Asian Cardiovasc Thorac Ann*. Jul 2016;24(6):546-54. doi:10.1177/0218492316653557
159. Martí JFP, Martín MÁP, Fernández-Villalba E, José PB. Approach to the development of a frailty index based on comprehensive geriatric assessment in nursing home. Article. *Farmacia Hospitalaria*. 2018;42(4):159-162. doi:10.7399/fh.10953
160. Martinez-Ramirez A, Martinikorena I, Gomez M, et al. Frailty assessment based on trunk kinematic parameters during walking. *J Neuroeng Rehabil*. May 24 2015;12:48. doi:10.1186/s12984-015-0040-6
161. Maxwell D, Rhee P, Drake M, Hodge J, Ingram W, Williams R. Development of the Burn Frailty Index: A prognostication index for elderly patients sustaining burn injuries. *Am J Surg*. Jul 2019;218(1):87-94. doi:10.1016/j.amjsurg.2018.11.012
162. McCarthy AL, Peel NM, Gillespie KM, et al. Validation of a frailty index in older cancer patients with solid tumours. *BMC Cancer*. Sep 14 2018;18(1):892. doi:10.1186/s12885-018-4807-6
163. McIsaac DI, Harris EP, Hladkowicz E, et al. Prospective Comparison of Preoperative Predictive Performance Between 3 Leading Frailty Instruments. Article. *Anesthesia and Analgesia*. 2020;131(1):263-272. doi:10.1213/ANE.0000000000004475
164. McIsaac DI, Taljaard M, Bryson GL, et al. Comparative assessment of two frailty instruments for risk-stratification in elderly surgical patients: study protocol for a prospective cohort study. *BMC Anesthesiol*. Nov 14 2016;16(1):111. doi:10.1186/s12871-016-0276-0
165. McIsaac DI, Wong CA, Huang A, Moloo H, van Walraven C. Derivation and Validation of a Generalizable Preoperative Frailty Index Using Population-based Health Administrative Data. *Ann Surg*. Jul 2019;270(1):102-108. doi:10.1097/SLA.0000000000002769
166. Meng Y, Zhao P, Yong R. Modified Frailty Index Independently Predicts Postoperative Pulmonary Infection in Elderly Patients Undergoing Radical Gastrectomy for Gastric Cancer. *Cancer Manag Res*. 2021;13:9117-9126. doi:10.2147/CMAR.S336023
167. Meulendijks FG, Hamaker ME, Boereboom FT, Kalf A, Vogtlander NP, van Munster BC. Groningen frailty indicator in older patients with end-stage renal disease. *Ren Fail*. 2015;37(9):1419-24. doi:10.3109/0886022X.2015.1077315
168. Mian HS, Wildes TM, Fiala MA. Development of a Medicare Health Outcomes Survey Deficit-Accumulation Frailty Index and Its Application to Older Patients With Newly Diagnosed Multiple Myeloma. *JCO Clin Cancer Inform*. 2018;2doi:10.1200/CCI.18.00043
169. Minici D, Cola G, Giordano A, et al. Towards Automated Assessment of Frailty Status Using a Wrist-Worn Device. *IEEE J Biomed Health Inform*. Mar 2022;26(3):1013-1022. doi:10.1109/JBHI.2021.3100979
170. Minici D, Cola G, Giordano A, et al. Wavelet-based analysis of gait for automated frailty assessment with a wrist-worn device. 2021:
171. Mulasso A, Roppolo M, Gobbens RJ, Rabaglietti E. The Italian Version of the Tilburg Frailty Indicator: Analysis of Psychometric Properties. *Res Aging*. Nov 2016;38(8):842-63. doi:10.1177/0164027515606192
172. Mulasso A, Roppolo M, Gobbens RJJ, Rabaglietti E. The evaluation of frailty in older adults: Translation and cross-cultural adaptation of the Tilburg Frailty Indicator to the Italian context. Article. *Giornale Italiano di Psicologia*. 2015;42(3):593-615. doi:10.1421/81168
173. Mulasso A, Roppolo M, Gobbens RJJ, Rabaglietti E. The Italian Version of the Tilburg Frailty Indicator: Analysis of Psychometric Properties. Article. *Research on Aging*. 2016;38(8):842-863. doi:10.1177/0164027515606192
174. Mullen MM, McKinnish TR, Fiala MA, et al. A deficit-accumulation frailty index predicts survival outcomes in patients with gynecologic malignancy. *Gynecol Oncol*. Jun 2021;161(3):700-704. doi:10.1016/j.ygyno.2021.02.027
175. Munguia L, Solis V, Meaney E, et al. Association of physical performance tests with frailty indicators and oxidative stress markers in a sample of a community-dwelling elderly population. Article. *Biomedical Research (India)*. 2018;29(17):3344-3350. doi:10.4066/biomedicalresearch.29-18-806
176. Nct. A Personalised Approach Utilising the Frailty Index to Empower Consumers. Trial registry record; Clinical trial protocol. *https://clinicaltrialsgov/show/NCT05292989*. 2022;
177. Negrete-Najar JP, Sehovic M, Rodriquenz MG, Garcia-Martinez J, Extermann M. Development of a health data derived frailty index as a predictor of adverse outcomes in older patients with pancreatic cancer. *J Geriatr Oncol*. Apr 2022;13(3):308-314. doi:10.1016/j.jgo.2021.10.009
178. Nguyen QD, Moodie EM, Keezer MR, Wolfson C. Clinical Correlates and Implications of the Reliability of the Frailty Index in the Canadian Longitudinal Study on Aging. *J Gerontol A Biol Sci Med Sci*. Oct 13 2021;76(11):e340-e346. doi:10.1093/gerona/glab161
179. Nishijima TF, Esaki T, Morita M, Toh Y. Preoperative frailty assessment with the Robinson Frailty Score, Edmonton Frail Scale, and G8 and adverse postoperative outcomes in older surgical patients with cancer. *Eur J Surg Oncol*. Apr 2021;47(4):896-901. doi:10.1016/j.ejso.2020.09.031
180. Nishijima TF, Shimokawa M, Esaki T, Morita M, Toh Y, Muss HB. A 10-Item Frailty Index Based on a Comprehensive Geriatric Assessment (FI-CGA-10) in Older Adults with Cancer: Development and Construct Validation. *Oncologist*. Oct 2021;26(10):e1751-e1760. doi:10.1002/onco.13894
181. Nowak W, Kowalik I, Kuzin M, et al. Comparison of the prognostic value of frailty assessment tools in patients aged >/= 65 years hospitalized in a cardiac care unit with acute coronary syndrome. *J Geriatr Cardiol*. May 28 2022;19(5):343-353. doi:10.11909/j.issn.1671-5411.2022.05.010
182. O'Donoghue PJ, Claffey P, Rice C, et al. Association between gait speed and the SHARE Frailty Instrument in a Falls and Syncope Clinic. *Eur Geriatr Med*. Oct 2021;12(5):1101-1105. doi:10.1007/s41999-021-00509-0
183. O'Donovan M, Sezgin D, Kabir Z, Liew A, O'Caoimh R. Assessing Global Frailty Scores: Development of a Global Burden of Disease-Frailty Index (GBD-FI). *Int J Environ Res Public Health*. Aug 6 2020;17(16)doi:10.3390/ijerph17165695
184. Oetsma S, Boonen A, Starmans M, Peeters R, van Onna M. Validation of two frailty questionnaires in older patients with rheumatoid arthritis: A cross-sectional study. Article. *Clinical and Experimental Rheumatology*. 2020;38(3):523-528.
185. Oh E, Hong GRS. The evaluation of feasibility and predictive validity of comprehensive korean frailty instrument: Using the 2008 and 2011 living profiles of older people survey in Korea. Article. *Journal of Korean Academy of Community Health Nursing*. 2017;28(2):206-215. doi:10.12799/jkachn.2017.28.2.206
186. Olivieri-Mui BL, Shi SM, McCarthy EP, Habtemariam D, Kim DH. Beyond the Health Deficit Count: Examining Deficit Patterns in a Deficit-Accumulation Frailty Index. *J Am Geriatr Soc*. Mar 2021;69(3):792-797. doi:10.1111/jgs.16955
187. Ondeck NT, Bovonratwet P, Ibe IK, et al. Discriminative Ability for Adverse Outcomes After Surgical Management of Hip Fractures: A Comparison of the Charlson Comorbidity Index, Elixhauser Comorbidity Measure, and Modified Frailty Index. *J Orthop Trauma*. May 2018;32(5):231-237. doi:10.1097/BOT.0000000000001140
188. Orfila F, Carrasco-Ribelles LA, Abellana R, et al. Validation of an electronic frailty index with electronic health records: eFRAGICAP index. *BMC Geriatr*. May 7 2022;22(1):404. doi:10.1186/s12877-022-03090-8
189. Orkaby AR, James K, Leuchtenburg J, Solooki E, Gaziano JM, Driver JA. Taking prevention to the next step: implementation of a brief, sustainable frailty assessment in a cardiology clinic. *BMJ Open Qual*. Jan 2021;10(1)doi:10.1136/bmjoq-2020-001140
190. Osaki T, Saito H, Shimizu S, et al. Modified Frailty Index is Useful in Predicting Non-home Discharge in Elderly Patients with Gastric Cancer Who Undergo Gastrectomy. *World J Surg*. Nov 2020;44(11):3837-3844. doi:10.1007/s00268-020-05691-z
191. Oviedo-Briones M, Laso AR, Carnicero JA, et al. A Comparison of Frailty Assessment Instruments in Different Clinical and Social Care Settings: The Frailtools Project. *J Am Med Dir Assoc*. Mar 2021;22(3):607 e7-607 e12. doi:10.1016/j.jamda.2020.09.024
192. Oviedo-Briones M, Rodríguez-Laso Á, Carnicero JA, et al. The ability of eight frailty instruments to identify adverse outcomes across different settings: the FRAILTOOLS project. Article. *Journal of Cachexia, Sarcopenia and Muscle*. 2022;13(3):1487-1501. doi:10.1002/jcsm.12990
193. Owodunni OP, Mostales JC, Qin CX, Gabre-Kidan A, Magnuson T, Gearhart SL. Preoperative Frailty Assessment, Operative Severity Score, and Early Postoperative Loss of Independence in Surgical Patients Age 65 Years or Older. *J Am Coll Surg*. Apr 2021;232(4):387-395. doi:10.1016/j.jamcollsurg.2020.11.026
194. Paiella S, De Pastena M, Esposito A, et al. Modified Frailty Index to Assess Risk in Elderly Patients Undergoing Distal Pancreatectomy: A Retrospective Single-Center Study. *World J Surg*. Apr 2022;46(4):891-900. doi:10.1007/s00268-021-06436-2
195. Park CM, Kim W, Lee ES, et al. Comparison of Frailty Index to Pneumonia Severity Measures in Older Patients With Pneumonia. *J Am Med Dir Assoc*. Jan 2022;23(1):165-169. doi:10.1016/j.jamda.2021.08.044
196. Paulson D, Lichtenberg PA. The Paulson-Lichtenberg Frailty Index: evidence for a self-report measure of frailty. *Aging Ment Health*. 2015;19(10):892-901. doi:10.1080/13607863.2014.986645
197. Peng LN, Hsiao FY, Lee WJ, Huang ST, Chen LK. Comparisons Between Hypothesis- and Data-Driven Approaches for Multimorbidity Frailty Index: A Machine Learning Approach. *J Med Internet Res*. Jun 11 2020;22(6):e16213. doi:10.2196/16213
198. Pereira AA, Borim FSA, Neri AL. Absence of association between frailty index and survival in elderly Brazilians: the FIBRA Study. *Cad Saude Publica*. Jun 12 2017;33(5):e00194115. Ausencia de associacao entre o indice de fragilidade e a sobrevivencia de idosos no Brasil: Estudo FIBRA. doi:10.1590/0102-311X00194115
199. Peris-Marti JF, Parro Martin MA, Fernandez-Villalba E, Bravo Jose P. Approach to the development of a frailty index based on comprehensive geriatric assessment in nursing home. *Farm Hosp*. Jul 1 2018;42(4):159-162. Aproximacion al desarrollo de un indice de fragilidad basado en la valoracion integral geriatrica en centros sociosanitarios. doi:10.7399/fh.10953
200. Perna S, Francis MD, Bologna C, et al. Performance of Edmonton Frail Scale on frailty assessment: its association with multi-dimensional geriatric conditions assessed with specific screening tools. *BMC Geriatr*. Jan 4 2017;17(1):2. doi:10.1186/s12877-016-0382-3
201. Peters LL, Boter H, Burgerhof JG, Slaets JP, Buskens E. Construct validity of the Groningen Frailty Indicator established in a large sample of home-dwelling elderly persons: Evidence of stability across age and gender. *Exp Gerontol*. Sep 2015;69:129-41. doi:10.1016/j.exger.2015.05.006
202. Peters R, Beckett N, Warwick J, et al. Laboratory tests combined into a frailty index predict mortality and cardiovascular events in hypertensive older adults. Journal article; Conference proceeding. *European heart journal*. 2015;36:978‐979. doi:10.1093/eurheartj/ehv401
203. Pizzonia M, Giannotti C, Carmisciano L, et al. Frailty assessment, hip fracture and long-term clinical outcomes in older adults. *Eur J Clin Invest*. Apr 2021;51(4):e13445. doi:10.1111/eci.13445
204. Pradeep Kumar D, Toosizadeh N, Mohler J, Ehsani H, Mannier C, Laksari K. Sensor-based characterization of daily walking: a new paradigm in pre-frailty/frailty assessment. *BMC Geriatr*. May 6 2020;20(1):164. doi:10.1186/s12877-020-01572-1
205. Pradhananga S, Regmi K, Razzaq N, Ettefaghian A, Dey AB, Hewson D. Ethnic differences in the prevalence of frailty in the United Kingdom assessed using the electronic Frailty Index. *Aging Med (Milton)*. Sep 2019;2(3):168-173. doi:10.1002/agm2.12083
206. Raj A, Nath RK, Pandit BN, Singh AP, Pandit N, Aggarwal P. Comparing the Modified Frailty Index with conventional scores for prediction of cardiac resynchronization therapy response in patients with heart failure. *J Frailty Sarcopenia Falls*. Jun 2021;6(2):79-85. doi:10.22540/JFSF-06-079
207. Ravensbergen WM, Blom JW, Evers AW, Numans ME, de Waal MW, Gussekloo J. Measuring daily functioning in older persons using a frailty index: a cohort study based on routine primary care data. *Br J Gen Pract*. Dec 2020;70(701):e866-e873. doi:10.3399/bjgp20X713453
208. Rejeski J, Xiao T, Wheless W, Pajewski NM, Jensen E, Callahan KE. An automated electronic health-record derived frailty index is associated with adverse events after endoscopy. *J Am Geriatr Soc*. Feb 2022;70(2):629-631. doi:10.1111/jgs.17537
209. Ritt M, Bollheimer LC, Sieber CC, Gaßmann KG. Prediction of one-year mortality by five different frailty instruments: A comparative study in hospitalized geriatric patients. Article. *Archives of Gerontology and Geriatrics*. 2016;66:66-72. doi:10.1016/j.archger.2016.05.004
210. Ritt M, Jager J, Ritt JI, Sieber CC, Gassmann KG. Operationalizing a frailty index using routine blood and urine tests. *Clin Interv Aging*. 2017;12:1029-1040. doi:10.2147/CIA.S131987
211. Ritt M, Radi KH, Schwarz C, Bollheimer LC, Sieber CC, Gassmann KG. A comparison of Frailty Indexes Based on a Comprehensive Geriatric Assessment for the Prediction of Adverse Outcomes. *J Nutr Health Aging*. 2016;20(7):760-7. doi:10.1007/s12603-015-0644-3
212. Rittberg R, Zhang H, Lambert P, Kudlovich R, Kim CA, Dawe DE. Utility of the modified frailty index in predicting toxicity and cancer outcomes for older adults with advanced pancreatic cancer receiving first-line palliative chemotherapy. *J Geriatr Oncol*. Jan 2021;12(1):112-117. doi:10.1016/j.jgo.2020.07.004
213. Rockwood K, McMillan M, Mitnitski A, Howlett SE. A Frailty Index Based on Common Laboratory Tests in Comparison With a Clinical Frailty Index for Older Adults in Long-Term Care Facilities. *J Am Med Dir Assoc*. Oct 1 2015;16(10):842-7. doi:10.1016/j.jamda.2015.03.027
214. Romeo FJ, Smietniansky M, Cal M, et al. Measuring frailty in patients with severe aortic stenosis: a comparison of the edmonton frail scale with modified fried frailty assessment in patients undergoing transcatheter aortic valve replacement. *J Geriatr Cardiol*. Jul 28 2020;17(7):441-446. doi:10.11909/j.issn.1671-5411.2020.07.001
215. Ryan J, Espinoza S, Ernst ME, et al. Validation of a Deficit-Accumulation Frailty Index in the ASPirin in Reducing Events in the Elderly Study and Its Predictive Capacity for Disability-Free Survival. *J Gerontol A Biol Sci Med Sci*. Jan 7 2022;77(1):19-26. doi:10.1093/gerona/glab225
216. Sacco R, Condoluci A, Curto LS, et al. A new frailty index as a risk predictor of morbidity and mortality: Its application in a surgery unit. Article. *European Journal of Oncology*. 2018;23(1):41-46.
217. Sacha M, Sacha J, Wieczorowska-Tobis K. Simultaneous Employment of the FRAIL Scale and the Tilburg Frailty Indicator May Identify Elderly People Who Require Different Interventional Strategies. *Clin Interv Aging*. 2020;15:683-690. doi:10.2147/CIA.S250437
218. Saricaoğlu F, Aksoy ŞM, Yilmazlar A, et al. Predicting mortality and morbidity of geriatric femoral fractures using a modified frailty index and perioperative features: A prospective, multicentre and observational study. Article. *Turk Geriatri Dergisi*. 2018;21(2):118-127. doi:10.31086/tjgeri.2018240413
219. Schuijt HJ, Morin ML, Allen E, Weaver MJ. Does the frailty index predict discharge disposition and length of stay at the hospital and rehabilitation facilities? *Injury*. Jun 2021;52(6):1384-1389. doi:10.1016/j.injury.2021.01.004
220. Schülein S, Sieber CC, Gaßmann KG, Ritt M. Frail older individuals maintaining a steady standing position: Associations between sway measurements with frailty status across four different frailty instruments. Article. *Clinical Interventions in Aging*. 2020;15:451-467. doi:10.2147/CIA.S223056
221. Segal JB, Chang HY, Du Y, Walston JD, Carlson MC, Varadhan R. Development of a Claims-based Frailty Indicator Anchored to a Well-established Frailty Phenotype. *Med Care*. Jul 2017;55(7):716-722. doi:10.1097/MLR.0000000000000729
222. Segal JB, Huang J, Roth DL, Varadhan R. External validation of the claims-based frailty index in the national health and aging trends study cohort. *Am J Epidemiol*. Sep 15 2017;186(6):745-747. doi:10.1093/aje/kwx257
223. Semmarath W, Seesen M, Yodkeeree S, et al. The association between frailty indicators and blood-based biomarkers in early-old community dwellers of Thailand. Article. *International Journal of Environmental Research and Public Health*. 2019;16(18)3457. doi:10.3390/ijerph16183457
224. Sentandreu-Mañó T, Fernández I, Cebrià i Iranzo MÀ, Tomás JM. Dimensions underlying frailty indicators in the Kihon Checklist. Article. *Geriatrics and Gerontology International*. 2019;19(10):982-987. doi:10.1111/ggi.13754
225. Shah SP, Penn K, Kaplan SJ, et al. Comparison of bedside screening methods for frailty assessment in older adult trauma patients in the emergency department. *Am J Emerg Med*. Jan 2019;37(1):12-18. doi:10.1016/j.ajem.2018.04.028
226. Shahrokni A, Tin A, Alexander K, et al. Development and Evaluation of a New Frailty Index for Older Surgical Patients With Cancer. *JAMA Netw Open*. May 3 2019;2(5):e193545. doi:10.1001/jamanetworkopen.2019.3545
227. Shi GP, Ma T, Zhu YS, et al. Frailty phenotype, frailty index and risk of mortality in Chinese elderly population- Rugao longevity and ageing study. *Arch Gerontol Geriatr*. Jan - Feb 2019;80:115-119. doi:10.1016/j.archger.2018.11.001
228. Shi S, Afilalo J, Lipsitz LA, et al. Frailty Phenotype and Deficit Accumulation Frailty Index in Predicting Recovery After Transcatheter and Surgical Aortic Valve Replacement. *J Gerontol A Biol Sci Med Sci*. Jul 12 2019;74(8):1249-1256. doi:10.1093/gerona/gly196
229. Shi SM, McCarthy EP, Mitchell S, Kim DH. Changes in Predictive Performance of a Frailty Index with Availability of Clinical Domains. *J Am Geriatr Soc*. Aug 2020;68(8):1771-1777. doi:10.1111/jgs.16436
230. Shi SM, McCarthy EP, Mitchell SL, Kim DH. Predicting Mortality and Adverse Outcomes: Comparing the Frailty Index to General Prognostic Indices. *J Gen Intern Med*. May 2020;35(5):1516-1522. doi:10.1007/s11606-020-05700-w
231. Shi SM, Olivieri-Mui B, McCarthy EP, Kim DH. Changes in a Frailty Index and Association with Mortality. *J Am Geriatr Soc*. Apr 2021;69(4):1057-1062. doi:10.1111/jgs.17002
232. Sison SDM, Newmeyer N, Arias KT, et al. Feasibility of implementing a telephone-based frailty assessment. *J Am Geriatr Soc*. Sep 28 2022;doi:10.1111/jgs.18031
233. Sohn B, Choi JW, Hwang HY, Jang MJ, Kim KH, Kim KB. Frailty Index is Associated with Adverse Outcomes after Aortic Valve Replacement in Elderly Patients. *J Korean Med Sci*. Aug 12 2019;34(31):e205. doi:10.3346/jkms.2019.34.e205
234. Soldati A, Poggi MM, Azzolino D, Vettoretti S, Cesari M. Frailty index and adverse outcomes in older patients in haemodialysis. *Arch Gerontol Geriatr*. Jul-Aug 2022;101:104673. doi:10.1016/j.archger.2022.104673
235. Soler V, Sourdet S, Balardy L, et al. Visual Impairment Screening at the Geriatric Frailty Clinic for Assessment of Frailty and Prevention of Disability at the Gerontopole. *J Nutr Health Aging*. 2016;20(8):870-877. doi:10.1007/s12603-015-0648-z
236. Solla-Suarez P, Avanzas P, Pascual I, et al. Frailty Assessment in a Cohort of Elderly Patients with Severe Symptomatic Aortic Stenosis: Insights from the FRailty Evaluation in Severe Aortic Stenosis (FRESAS) Registry. *J Clin Med*. May 27 2021;10(11)doi:10.3390/jcm10112345
237. Stolz E, Mayerl H, Hoogendijk EO, Armstrong JJ, Roller-Wirnsberger R, Freidl W. Acceleration of health deficit accumulation in late-life: evidence of terminal decline in frailty index three years before death in the US Health and Retirement Study. *Ann Epidemiol*. Jun 2021;58:156-161. doi:10.1016/j.annepidem.2021.03.008
238. Stolz E, Rasky E, Jagsch C. Frailty index predicts geriatric psychiatry inpatient mortality: a case-control study. *Psychogeriatrics*. Jul 2020;20(4):469-472. doi:10.1111/psyg.12535
239. Strom JB, Xu J, Orkaby AR, et al. Identification of Frailty Using a Claims-Based Frailty Index in the CoreValve Studies: Findings from the EXTEND-FRAILTY Study. *J Am Heart Assoc*. Oct 5 2021;10(19):e022150. doi:10.1161/JAHA.121.022150
240. Subramaniam S, Aalberg JJ, Soriano RP, Divino CM. The 5-Factor Modified Frailty Index in the Geriatric Surgical Population. *Am Surg*. Sep 2021;87(9):1420-1425. doi:10.1177/0003134820952438
241. Tamura K, Matsuda K, Fujita Y, et al. Optimal Assessment of Frailty Predicts Postoperative Complications in Older Patients with Colorectal Cancer Surgery. *World J Surg*. Apr 2021;45(4):1202-1209. doi:10.1007/s00268-020-05886-4
242. Tanaka S, Kamiya K, Hamazaki N, et al. Incremental Value of Objective Frailty Assessment to Predict Mortality in Elderly Patients Hospitalized for Heart Failure. *J Card Fail*. Nov 2018;24(11):723-732. doi:10.1016/j.cardfail.2018.06.006
243. Tanaka S, Ueno M, Iida H, et al. Preoperative assessment of frailty predicts age-related events after hepatic resection: a prospective multicenter study. *J Hepatobiliary Pancreat Sci*. Aug 2018;25(8):377-387. doi:10.1002/jhbp.568
244. Tanaka T, Hirano H, Ohara Y, Nishimoto M, Iijima K. Oral Frailty Index-8 in the risk assessment of new-onset oral frailty and functional disability among community-dwelling older adults. *Arch Gerontol Geriatr*. May-Jun 2021;94:104340. doi:10.1016/j.archger.2021.104340
245. Tanaka T, Talegawkar SA, Jin Y, Bandinelli S, Ferrucci L. Association of Adherence to the Mediterranean-Style Diet with Lower Frailty Index in Older Adults. *Nutrients*. Mar 30 2021;13(4)doi:10.3390/nu13041129
246. Tanaka T, Talegawkar SA, Jin Y, et al. Metabolomic Profile of Different Dietary Patterns and Their Association with Frailty Index in Community-Dwelling Older Men and Women. *Nutrients*. May 27 2022;14(11)doi:10.3390/nu14112237
247. Theou O, Tan EC, Bell JS, et al. Frailty Levels in Residential Aged Care Facilities Measured Using the Frailty Index and FRAIL-NH Scale. *J Am Geriatr Soc*. Nov 2016;64(11):e207-e212. doi:10.1111/jgs.14490
248. Theou O, van der Valk AM, Godin J, et al. Exploring Clinically Meaningful Changes for the Frailty Index in a Longitudinal Cohort of Hospitalized Older Patients. *J Gerontol A Biol Sci Med Sci*. Sep 25 2020;75(10):1928-1934. doi:10.1093/gerona/glaa084
249. Thompson MQ, Theou O, Yu S, Adams RJ, Tucker GR, Visvanathan R. Frailty prevalence and factors associated with the Frailty Phenotype and Frailty Index: Findings from the North West Adelaide Health Study. *Australas J Ageing*. Jun 2018;37(2):120-126. doi:10.1111/ajag.12487
250. Tong T, Chignell M, Tierney MC, et al. Tablet-based frailty assessments in emergency care for older adults. 2016:613-617.
251. Toosizadeh N, Eskandari M, Ehsani H, Parvaneh S, Asghari M, Sweitzer N. Frailty assessment using a novel approach based on combined motor and cardiac functions: a pilot study. *BMC Geriatr*. Mar 14 2022;22(1):199. doi:10.1186/s12877-022-02849-3
252. Topcu Y, Tufan F, Kilic C. Turkish version of the Tilburg Frailty Indicator. *Clin Interv Aging*. 2019;14:615-620. doi:10.2147/CIA.S197512
253. Tournas G, Kourek C, Mantzaraki V, et al. Assessment of frailty and related outcomes in older patients with heart failure: A cohort study. *Hellenic J Cardiol*. Sep-Oct 2022;67:42-47. doi:10.1016/j.hjc.2022.04.004
254. Uchmanowicz I, Lisiak M, Wontor R, Loboz-Grudzien K. Frailty in patients with acute coronary syndrome: comparison between tools for comprehensive geriatric assessment and the Tilburg Frailty Indicator. *Clin Interv Aging*. 2015;10:521-9. doi:10.2147/CIA.S78365
255. Uchmanowicz I, Lomper K, Gros M, et al. Assessment of Frailty and Occurrence of Anxiety and Depression in Elderly Patients with Atrial Fibrillation. *Clin Interv Aging*. 2020;15:1151-1161. doi:10.2147/CIA.S258634
256. Vainqueur L, Simo-Tabue N, Villeneuve R, et al. Frailty index, mortality, and length of stay in a geriatric short-stay unit in Guadeloupe. *Front Med (Lausanne)*. 2022;9:963687. doi:10.3389/fmed.2022.963687
257. van Assen MA, Pallast E, Fakiri FE, Gobbens RJ. Measuring frailty in Dutch community-dwelling older people: Reference values of the Tilburg Frailty Indicator (TFI). *Arch Gerontol Geriatr*. Nov-Dec 2016;67:120-9. doi:10.1016/j.archger.2016.07.005
258. van Dam CS, Trappenburg MC, ter Wee MM, et al. The Accuracy of Four Frequently Used Frailty Instruments for the Prediction of Adverse Health Outcomes Among Older Adults at Two Dutch Emergency Departments: Findings of the AmsterGEM Study. Article. *Annals of Emergency Medicine*. 2021;78(4):538-548. doi:10.1016/j.annemergmed.2021.04.027
259. Van Der Ploeg E, Van Der Velde Y, Gobbens R. Predictive value of gait speed and frailty indicators on survival time and time until discharge to home in ambulant older patients in a specialized rehabilitation center. Article. *Journal of Geriatric Physical Therapy*. 2017;40(2):95-101. doi:10.1519/JPT.0000000000000074
260. van der Ploeg T, Gobbens R. A Comparison of Different Modeling Techniques in Predicting Mortality With the Tilburg Frailty Indicator: Longitudinal Study. *JMIR Med Inform*. Mar 30 2022;10(3):e31480. doi:10.2196/31480
261. Vrotsou K, Machon M, Rivas-Ruiz F, et al. Psychometric properties of the Tilburg Frailty Indicator in older Spanish people. *Arch Gerontol Geriatr*. Sep - Oct 2018;78:203-212. doi:10.1016/j.archger.2018.05.024
262. Waduud MA, Giannoudi M, Drozd M, et al. Morphometric and traditional frailty assessment in transcatheter aortic valve implantation. *J Cardiovasc Med (Hagerstown)*. Oct 2020;21(10):779-786. doi:10.2459/JCM.0000000000001014
263. Wang ZD, Yao S, Shi GP, et al. Frailty index is associated with increased risk of elevated BNP in an elderly population: the Rugao Longevity and Ageing Study. *Aging Clin Exp Res*. Feb 2020;32(2):305-311. doi:10.1007/s40520-019-01189-4
264. Wen YC, Chen LK, Hsiao FY. Predicting mortality and hospitalization of older adults by the multimorbidity frailty index. *PLoS One*. 2017;12(11):e0187825. doi:10.1371/journal.pone.0187825
265. Wilson S, Sutherland E, Razak A, et al. Implementation of a Frailty Assessment and Targeted Care Interventions and Its Association with Reduced Postoperative Complications in Elderly Surgical Patients. *J Am Coll Surg*. Dec 2021;233(6):764-775 e1. doi:10.1016/j.jamcollsurg.2021.08.677
266. Won CW, Lee Y, Lee S, Kim M. Development of Korean Frailty Index for Primary Care (KFI-PC) and Its Criterion Validity. *Ann Geriatr Med Res*. Jun 2020;24(2):125-138. doi:10.4235/agmr.20.0021
267. Wu S, Mulcahy J, Kasper JD, Kan HJ, Weiner JP. Comparing Survey-Based Frailty Assessment to Medicare Claims in Predicting Health Outcomes and Utilization in Medicare Beneficiaries. *J Aging Health*. Aug-Sep 2020;32(7-8):764-777. doi:10.1177/0898264319851995
268. Yamaguchi M, Yamada Y, Nanri H, et al. Association between the Frequency of Protein-Rich Food Intakes and Kihon-Checklist Frailty Indices in Older Japanese Adults: The Kyoto-Kameoka Study. *Nutrients*. Jan 13 2018;10(1)doi:10.3390/nu10010084
269. Yamashita S, Mashima N, Higuchi M, et al. Modified 5-Item Frailty Index Score as Prognostic Marker After Radical Cystectomy in Bladder Cancer. *Clin Genitourin Cancer*. Jun 2022;20(3):e210-e216. doi:10.1016/j.clgc.2021.12.016
270. Yang F, Gu D. Predictability of frailty index and its components on mortality in older adults in China. *BMC Geriatr*. Jul 25 2016;16:145. doi:10.1186/s12877-016-0317-z
271. Yao SM, Zheng PP, Wan YH, et al. Adding high-sensitivity C-reactive protein to frailty assessment to predict mortality and cardiovascular events in elderly inpatients with cardiovascular disease. *Exp Gerontol*. Apr 2021;146:111235. doi:10.1016/j.exger.2021.111235
272. Zhang X, Tan SS, Bilajac L, et al. Reliability and Validity of the Tilburg Frailty Indicator in 5 European Countries. *J Am Med Dir Assoc*. Jun 2020;21(6):772-779 e6. doi:10.1016/j.jamda.2020.03.019
273. Zhao F, Tang B, Liu X, et al. Development and validation of the geriatric trauma frailty index for geriatric trauma patients based on electronic hospital records. *Age Ageing*. Jan 6 2022;51(1)doi:10.1093/ageing/afab186
274. Zhu Y, Liu Z, Wang Y, et al. Agreement between the frailty index and phenotype and their associations with falls and overnight hospitalizations. *Arch Gerontol Geriatr*. Sep-Oct 2016;66:161-5. doi:10.1016/j.archger.2016.06.004
275. Zygomalas AN, Katsanos K, Skroubis G. Minimally Invasive Management of Acute Cholecystitis and Frailty Assessment in Geriatric Patients. *Surg Laparosc Endosc Percutan Tech*. Dec 9 2021;32(1):119-123. doi:10.1097/SLE.0000000000001021
